# The TLR7/8 agonist R848 remodels tumor and host responses to promote survival in pancreatic cancer

**DOI:** 10.1038/s41467-019-12657-w

**Published:** 2019-10-15

**Authors:** Katherine A. Michaelis, Mason A. Norgard, Xinxia Zhu, Peter R. Levasseur, Shamilene Sivagnanam, Shannon M. Liudahl, Kevin G. Burfeind, Brennan Olson, Katherine R. Pelz, Diana M. Angeles Ramos, H. Carlo Maurer, Kenneth P. Olive, Lisa M. Coussens, Terry K. Morgan, Daniel L. Marks

**Affiliations:** 1 Medical Scientist Training Program, Oregon Health & Science University, Portland, OR USA; 2 Brenden-Colson Center for Pancreatic Care, Oregon Health & Science University, Portland, OR USA; 3 Papé Family Pediatric Research Institute, Oregon Health & Science University, Portland, OR USA; 4 Department of Computational Biology, Oregon Health & Science University, Portland, OR USA; 5 Department of Cell, Developmental and Cancer Biology, Knight Cancer Institute, Oregon Health & Science University, Portland, OR USA; 6 Departments of Medicine and Pathology and Cell Biology, Columbia University Medical Center, New York, NY USA; 7 Department of Pathology, Oregon Health & Science University, Portland, OR USA

**Keywords:** Cancer microenvironment, Pancreatic cancer, Immunotherapy, Feeding behaviour, Metabolic diseases

## Abstract

A priority in cancer research is to innovate therapies that are not only effective against tumor progression but also address comorbidities such as cachexia that limit quality and quantity of life. We demonstrate that TLR7/8 agonist R848 induces anti-tumor responses and attenuates cachexia in murine models of pancreatic ductal adenocarcinoma (PDAC). In vivo, tumors from two of three cell lines were R848-sensitive, resulting in smaller tumor mass, increased immune complexity, increased CD8^+^ T-cell infiltration and activity, and decreased Treg frequency. R848-treated mice demonstrated improvements in behavioral and molecular cachexia manifestations, resulting in a near-doubling of survival duration. Knockout mouse studies revealed that stromal, not neoplastic, TLR7 is requisite for R848-mediated responses. In patient samples, we found *Tlr7* is ubiquitously expressed in stroma across all stages of pancreatic neoplasia, but epithelial *Tlr7* expression is relatively uncommon. These studies indicate immune-enhancing approaches including R848 may be useful in PDAC and cancer-associated cachexia.

## Abstract (web summary)

In the treatment of pancreatic ductal adenocarcinoma (PDAC), comorbidities such as cachexia limit quality of life and survival. Here, the authors show TLR7/8 agonist R848 remodels host and tumour immune responses, promoting survival and attenuating cachexia in murine models of PDAC.

## Introduction

With recent advances in the field of cancer immunotherapy, a number of highly effective therapies were developed to enhance anti-tumor immunity. Some of these approaches, such as immune checkpoint inhibitors and adoptive cell transfer, already demonstrate benefit for a subset of cancer patients^1,2^. Unfortunately, despite these advances for many forms of cancer, immune therapy approaches for patients with pancreatic ductal adenocarcinoma (PDAC) are lacking. With a median survival of 6 months and a 5-year survival rate of 8%, outcomes for PDAC remain dismal and new therapies are urgently needed^3^. Numerous biological rationales are hypothesized as underlying barriers for lack of effective immunotherapy responses in PDAC, including that the tumor microenvironment in PDAC is profoundly immunosuppressive. Not only are immune cell infiltrates sparse in pancreatic cancer, but an abundant presence of T cell-suppressive myeloid cells are thought to interfere with all stages of anti-tumor immune responses^4,5^. These factors make it clear that to create successful immunotherapy approaches in PDAC, it is essential to modify the immune landscape of these tumors.

One promising strategy for remodeling tumor immune composition and functionality in PDAC is to inhibit tumor-promoting inflammatory signaling pathways common to myeloid and lymphoid cells, including Toll-like receptors (TLRs). Stimulation of TLRs not only results in changes to myeloid cell activity in the tumor microenvironment, but augments the activity and specificity of adaptive immunity. For example, recent research highlights that stimulation of TLR7, an endosomal ssRNA receptor traditionally associated with viral response, lowers PD-1 (programmed cell death protein 1) expression on T cells and enhances CD8^+^ T-cell cytotoxic responses^6^. TLR7 agonists include native ssRNAs and small molecules such as imidazoquinolines and benzazepines.^7^ Imidazoquinolines are structurally similar to the most-studied class of TLR7 agonist and include the Food and Drug Administration (FDA)-approved imiquimod and its more potent counterpart resiquimod (R848).^8^ Small-molecule TLR7 agonists already demonstrated success in dermatologic malignancies including basal cell carcinoma^9^, but will require further optimization for other types of cancer. Although TLR7 is abundantly expressed in pancreatic cancer lesions^10^, studies of therapeutic TLR7 stimulation in PDAC are lacking.

An additional unexplored question is how targeted immunotherapies compare with conventional chemotherapies in their effects on cachexia and treatment-associated toxicities. Cachexia is a common and devastating comorbidity of cancer that limits therapeutic options, decreases quality of life, and increases mortality risk^11,12^. This is a highly pertinent clinical problem for malignancies such as PDAC, in which up to 80% of patients suffer from cachexia^13,14^. Many chemotherapy agents induce or worsen cachexia by independently causing anorexia, weight loss, muscle wasting, and fatigue^15,16^. Acute systemic inflammatory responses, often elicited by immunotherapies, result in adverse events such as fever and fatigue^17^. In accordance with the idea that treatment outcomes are the sum of the treatment’s effects on tumor and host, it is crucial to evaluate the effects immunotherapeutic strategies exert both on the tumor and on host physiology.

In this study, we investigated whether the TLR7/8 agonist R848 elicits anti-tumor responses in syngeneic orthotopic (OT) murine PDAC models and whether this class of therapy is beneficial or harmful toward PDAC-associated cachexia. Moreover, we revealed how TLR7 agonists alter the tumor immune microenvironment using a combination of quantitative multiplex immunohistochemistry (mIHC) and flow cytometry. Behavioral and molecular phenotyping of cachexia status was performed for tumor-bearing animals to explore R848’s efficacy, and on tumor-naive animals to explore R848′s safety. After establishing evidence of benefit for both tumor burden and cachexia severity, we determined whether the effects of R848 in the tumor microenvironment were dependent on stromal or neoplastic cell TLR7. Finally, to explore therapeutic viability of R848 in human populations, we compared stromal and epithelial expression of *Tlr7* and related transcripts by RNA-sequencing (RNA-seq) in laser-capture microdissected human lesions across stages of pancreatic neoplasia.

## Results

### R848 reduces PDAC tumor burden and alters the tumor microenvironment

TLR agonists are employed for a variety of malignancies to induce anti-tumor immunity^9,18–20^, which we hypothesized could occur in the context of PDAC. Further, we hypothesized this response would depend on neoplastic epithelial cell factors regulating immune cell recruitment and neoantigen quality, both of which are necessary components of CD8^+^ T-cell-mediated anti-tumor immunity. To assess efficacy of R848 for induction of anti-tumor responses, animals were implanted with one of three KRAS^LSL.G12D/+^ P53^LSL.R172H/+^ Pdx-Cre (KPC)-derived neoplastic cell lines (KxPxCx, FC1199, FC1242) or given sham surgery (Sham). Each cell line was implanted into C57BL/6 mice using either atraumatic intraperitoneal (IP) or surgical OT routes, as a means of querying the role of pancreatic inflammation in drug response. Two days post-implantation, mice were randomized on the covariates of weight, body composition, and basal food intake, then were allocated to receive daily R848 or vehicle until study endpoint. For tumor response studies, the experimental endpoint for all groups was onset of end-stage cachexia or reaching maximum tumor burden in any experimental arm.

**Fig. 1:**
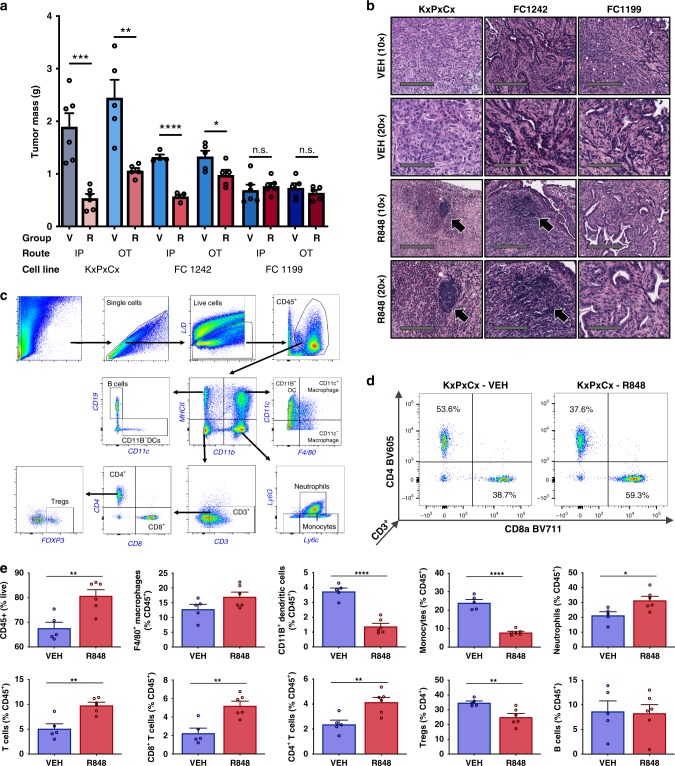
R848 effects on tumor mass and immune microenvironment. **a** Tumor mass in cell lines KxPxCx, FC1242, and FC1199, implanted either intraperitoneally (IP) or orthotopically (OT), allocated to vehicle (V) or R848 (R) treatment groups. **b** Representative H&E imaged at ×10 and ×20 magnification of KxPxCx, FC1242, and FC1199 OT tumors with and without R848 (scale bars represent 200 and 100 µm, respectively). Arrows indicate intratumoral lymphoid structures. **c** Gating strategy for quantification of immune cell complexity in tumors. **d** Representative plots from KxPxCx-engrafted animals treated with vehicle and R848, depicting CD4 and CD8 populations on flow cytometry. **e** Flow cytometry plots of R848-treated and -untreated KxPxCx tumor immune cells, *n* = 5–6/group. Data are depicted as mean ± SEM, with individual data points shown. Comparisons were performed as Student’s *t*-test. *P < 0.05; **P < 0.01; ***P < 0.001; ****P < 0.0001

Significant reductions in tumor mass were evident at endpoint in two of three KPC-derived cell lines, without sensitivity differences on the basis of implantation method (Fig. 1a). In the most R848-sensitive cell line, KxPxCx, anti-tumor response was more pronounced in IP implantation (71.7% reduction, *P* < 0.005) than OT implantation (56.6% reduction, *P* < 0.01). In the second R848-sensitive cell line, FC1242, sensitivity was also greater for IP implantation (57.0% reduction, *P* < 0.0001) than that of OT implantation (19.7% reduction, *P* < 0.05). No tumor mass differences were observed between R848 and vehicle treatment for cell line FC1199 with any method of implantation. For cell line KxPxCx, R848 resulted in decreased extra-pancreatic tumor growth, without gross evidence of invasion of lymph nodes, mesentery, liver, or other abdominal structures.

Histological analysis was performed on tumors at endpoint, with the goal of elucidating structural and cellular correlates for variations in R848 response. OT implantation of all cell lines produced pancreatic masses consistent with moderate-to-poorly differentiated PDAC (Fig. 1b). Tumors from KxPxCx and FC1242-implanted animals treated with R848 were characterized by increased lymphocytic infiltration and formation of intratumoral lymphoid structures (Fig. 1b, arrow). In contrast, histological examination of tumors derived from FC1199 did not show lymphoid structures or increased lymphocytic infiltrate in response to R848. These findings together indicated an epithelium-dependent role in immune cell recruitment and activity during R848 treatment.

Flow cytometry was used to compare tumor immune population frequencies between vehicle- and R848-treated KxPxCx tumors after 10 days of treatment (Fig. 1c). The overall frequency of CD45^+^ leukocytes was increased in R848-treated tumors, with widespread differences in both T lymphocyte and myeloid populations (Fig. 1d). The frequency of CD3^+^CD8^+^ T cells more than doubled following R848 treatment (Fig. 1d–e) (2.27% CD45^+^ (vehicle) vs. 5.23% CD45^+^ (R848), *P* < 0.01). CD3^+^CD4^+^ T-cell frequency increased following R848 treatment (2.39% CD45^+^ (vehicle) vs. 4.17% of CD45^+^ (R848), *P* < 0.01). Among CD3^+^CD4^+^ cells, the proportion of FOXP3^+^ regulatory T (Treg) cells significantly decreased following treatment with R848 (34.86% CD4^+^ (vehicle) vs. 25.07% (R848), *P* < 0.01) (Fig. 1e). Despite the presence of intratumoral lymphoid structures on histology, no overall differences were observed in B-cell frequency.

Among granulocytic populations, neutrophils were increased after treatment with R848 (21.48% of CD45^+^ events vehicle vs. 31.53% R848, *P* < 0.05). Both total macrophages (CD45^+^F4/80^+^) and CD11c^−^ macrophages trended toward increases with R848, but did not reach statistical significance. Following treatment with R848, both CD11b^+^ and CD11b^−^ dendritic cell (CD45^+^MHCII^+^CD11c^+^) frequency decreased (combined; 3.75% CD45^+^ events vehicle vs. 1.39% R848, *P* < 0.0001). Similarly, monocyte (CD45^+^MHCII^−^CD11b^+^) frequency decreased following treatment (24.12% of CD45^+^ vehicle vs. 7.98% of CD45^+^ R848, *P* < 0.0001).

**Fig. 2:**
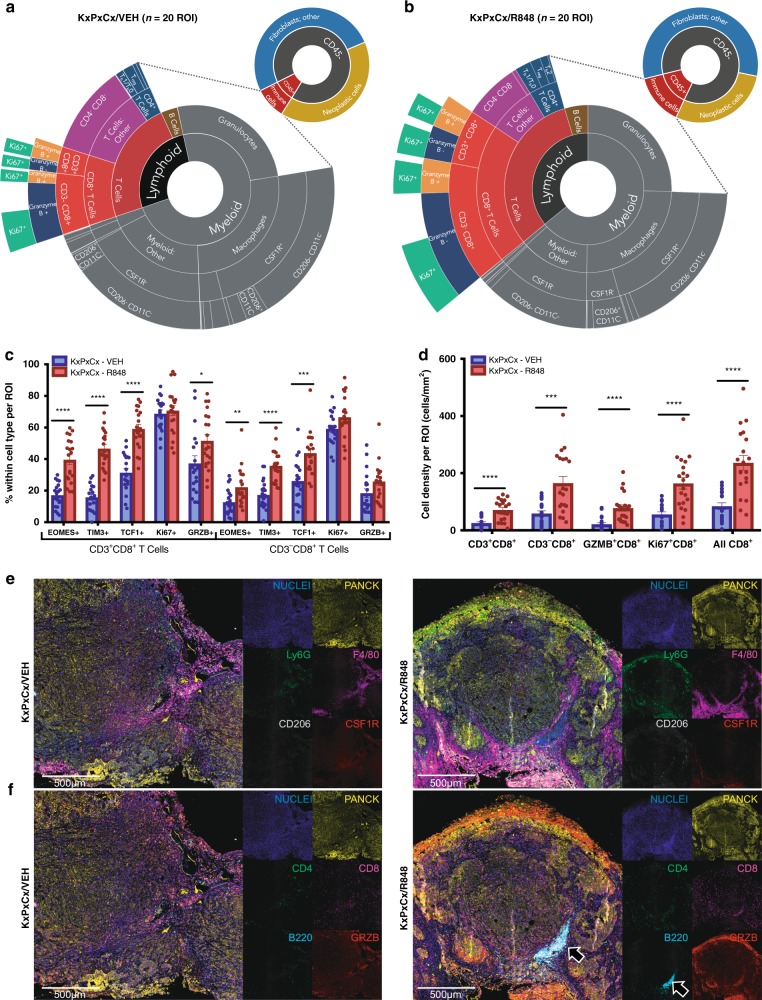
Tumor immune microenvironment and functional status are enhanced by R848. **a**, **b** Sunburst plots depicting image cytometry quantitation of tumor immune subsets for KxPxCx treated with vehicle (**a**) and KxPxCx treated with R848 (**b**). Upper right plots depict overall immune vs. non-immune composition within tumors, with lower left plots depicting immune composition and activation status. Plots represent four tumors per treatment group and five ROIs per tumor, representing a minimum of 50% of the tumor area. **c** Functional status analysis for both CD3+CD8+ and CD3-CD8+ T cells, including EOMES, TIM3, memory T-cell marker TCF1, proliferation and activity marker Ki67, and activity marker granzyme B (GRZB). **d** Cell density of overall, proliferative, and cytotoxic CD8+ T-cell populations per ROI in vehicle and R848-treated KxPxCx-derived tumors. **e** Representative myeloid multiplex imaging for vehicle and R848-treated tumors, emphasizing PANCK (tumor cells), F4/80 (macrophages), CD206 (tumor promotional marker), colony-stimulating factor 1 receptor (CSF1R; macrophage survival and differentiation), and Ly6G (granulocytes). **f** Representative lymphoid multiplex imaging for vehicle and R848-treated tumors, emphasizing PANCK, B220 (B-cell marker), CD4, CD8, and Granzyme B (cytotoxic activity marker). Scale bars represent 500 µm. Arrow indicates intratumoral lymphoid structure. Data are depicted as mean ±SEM, with individual data points shown. Comparisons are Student’s *T*-test, *P < 0.05; **P < 0.01; ***P < 0.001; ****P < 0.0001

For in-depth characterization of the tumor immune microenvironment, tissue sections from orthotopically implanted KxPxCx tumors treated with vehicle or R848 were analyzed in situ with a 23-marker quantitative mIHC (experimental pipeline and gating strategy described in Supplementary Fig. 1)^21^. This method permits quantification of the cellular composition of tumors with simultaneous spatial localization. This approach revealed that R848 resulted in overall increases in CD45^+^ cells throughout both edges and core of tumors, with broad changes in both immune cell complexity and functional status (Fig. 2a, b and Supplementary Figs. 2, 3).

In agreement with the flow cytometry analyses, R848 did not change the relative proportions of B cells (CD45^+^CD3^−^B220^+^) (2.83% CD45^+^ (vehicle) vs. 2.82% (R848)). However, although the majority of B cells were disseminated throughout tumor parenchyma in vehicle-treated tumors, R848-treated tumors revealed frequent formation of lymphoid aggregates (Fig. 2f and Supplementary Fig. 4). CD3^+^CD4^+^ T cells also evidenced generalized increases (0.27% total (vehicle) vs. 0.60% total (R848), *P* < 0.05), with significant changes to CD3^+^CD4^+^ subset frequency (Supplementary Fig. 2C). Consistent with the known mechanism of TLR7 agonists in promoting T_H_1 differentiation^22^, the most common CD3^+^CD4^+^ lineage in R848-treated tumors was GATA3^−^FOXP3^−^RORGT^−^, collectively representing T_H_0 and T_H_1 cells (34.11% CD3^+^CD4^+^ in vehicle vs. 41.86% in R848, *P* < 0.05). In contrast, the most common CD3^+^CD4^+^ subset in vehicle-treated tumors were Tregs (35.97% CD3^+^CD4^+^ cells in vehicle vs. 19.07% R848, *P* = 0.001).

Of importance to anti-neoplastic immunity, CD8^+^ T cells were more concentrated and distributed throughout the epithelial and stromal compartments following R848 treatment (Fig. 2c, d) (0.85% of total cells (vehicle) vs. 2.5% (R848), *P* < 0.0001). CD8^+^ T-cell density increased from 84.6 to 236.2 cells/mm^2^ following treatment with R848 (*P* < 0.0001) (Fig. 2d). In addition, granzyme B^+^ CD8^+^ T cells increased in density following treatment (21.62 cells/mm^2^ vehicle vs. 71.09 cells/mm^2^ R848, *P* < 0.0001), indicating improved cytotoxic effector function. R848 increased the cell density of proliferating CD8^+^ T cells as assessed by Ki67 (55.87 cells/mm^2^ vehicle vs. 164.1 R848, *P* < 0.0001). R848 also resulted in increased CD8^+^ T-cell expression of TIM3 (0.68% vehicle vs. 2.739% R848, *P* = 0.001) and EOMES (0.47% vehicle vs. 1.43% R848, *P* < 0.01), reflecting their varied differentiation and/or functional state. Within cell populations pertinent to T cell function, decreases in programmed cell death ligand-1 were observed among PANCK^+^ tumor cells (30.30% vehicle vs. 20.69% R848) and CD11b^+^ cells (16.20% vehicle vs. 12.83% R848); however, these changes did not approach statistical significance.

A sub-analysis of myeloid cells in CD45^+^ dense tumor regions demonstrated that in areas of immune cell enrichment, macrophages increased following R848 treatment from a mean of 0.55% to 8.08% of cells per region of interest (*P* < 0.01, *n* = 20 regions of interest (ROI)/group, Supplementary Fig. 3C). Macrophages following R848 treatment had relatively increased CSF1R positivity, but decreased CD206 positivity (Supplementary Fig. 3D). In CD45^+^ enriched regions, R848 also increased granulocyte infiltration from a mean of 1.93% to 4.41% (*P* < 0.01). However, dendritic cells were decreased from a mean of 1.5% CD45^+^ cells in vehicle-treated tumors to 0.54% CD45^+^ cells in R848-treated tumors (*P* < 0.001).

### R848 is well-tolerated and improves PDAC cachexia manifestations

**Fig. 3:**
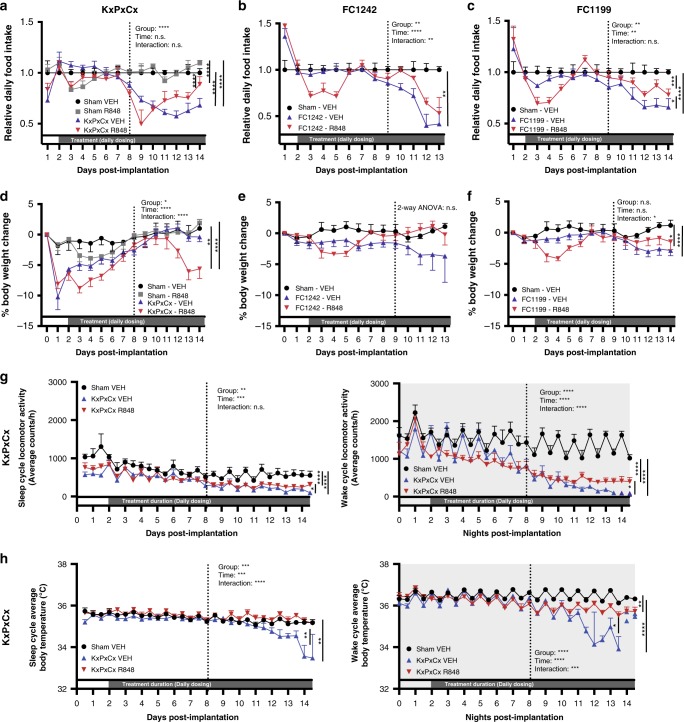
R848 improves cachexia manifestations in murine PDAC models. Mice were orthotopically implanted with one of three distinct syngeneic KPC-derived cell lines: KxPxCx, FC1199, and FC1242. Vertical dotted lines denote the onset of cachexia as defined by consistent hypophagia exceeding 10% below control food intake. KxPxCx studies were performed independently, whereas FC1242 and FC1199 studies were performed concurrently with a shared control group. **a** Food intake relative to sham-operated vehicle-treated mice (Sham-VEH) for sham-operated R848-treated mice (Sham-R848), KxPxCx-engrafted mice receiving vehicle (KxPxCx-VEH), and KxPxCx-engrafted mice receiving R848 (KxPxCx-R848). **b**, **c** Food intake relative to sham-operated vehicle-treated mice implanted for FC1242 (**b**) and FC1199 (**c**) treated with vehicle (VEH) or R848. **d**–**f** Body weight relative to baseline for KxPxCx (**d**), FC1242 (**e**), and FC1199 (**f**) throughout treatment with R848 or vehicle. **g** Average locomotor activity during day/sleep and night/wake cycles for KxPxCx-implanted animals treated with R848 or vehicle, binned into 6 h intervals. **h** Average body temperature during day/sleep and night/wake cycles for KxPxCx-implanted animals treated with R848 or vehicle, binned into 6 h intervals. Data for all panels are displayed as mean ± SEM, unless fewer than three subjects were remaining in a group, in which case individual data points are plotted. Statistics were performed as two-way ANOVA during the cachexia stage to test for main effects of tumor and treatment, and the interaction thereof, with multiple comparisons to test for main column effects. Comparisons are two-way ANOVA analyzing group by time, with multiple comparison analysis of the main column effect during cachexia stage. *P < 0.05; **P < 0.01; ***P < 0.001; ****P < 0.0001

Despite their beneficial effects on tumor response, significant concerns exist about TLR agonists’ potential systemic toxicities. Indeed, cancer cachexia is an often-deadly comorbidity and widely believed to be a disorder of inflammatory responses. Therefore, it is critical to ensure cachexia is not worsened by immunotherapies that alter systemic inflammatory signaling. To assess tolerability, mice were orthotopically implanted with one of three KPC-derived cell lines (KxPxCx, FC1199, FC1242) or given sham surgery (Sham), allocated to R848 or vehicle treatment, then tracked for food intake, body weight, locomotor activity, and body temperature (Fig. 3). Consistent with known effects of TLR agonists, R848 treatment induction resulted in a brief hypophagia and weight loss. Following this stage, R848-treated mice exhibited comparable food intake to vehicle-treated KPC-bearing mice in pre-cachexia stages of illness. However, ongoing R848 treatment increased cachexia stage food intake above that of vehicle-treated tumor-bearing animals in all three cell lines tested (Fig. 3a–c). KxPxCx-bearing mice treated with R848 initially developed significant abdominal distention and weight gain consistent with ascites relative to untreated KxPxCx-bearing animals, but underwent subsequent weight loss and regression of abdominal distention as treatment progressed (Fig. 3d). In contrast, FC1242 and FC1199 did not produce ascites, and mice receiving R848 were modestly protected from weight loss during later stages of illness (Fig. 3e–f). During pre-cachexia stage, KxPxCx mice treated with R848 exhibited treatment-related decreases in locomotion, but in later stages of illness exhibited improvements in locomotion relative to vehicle-treated subjects (Fig. 3g).

Although vehicle-treated KxPxCx mice became hypothermic during cachexia stages of illness, R848-treated KxPxCx mice were significantly less hypothermic throughout the study (Fig. 3h). Importantly, no fever or hypothermia was observed with R848 treatment alone. In sham-operated controls, R848 only resulted in transient decreases in body weight and appetite, suggesting that sickness responses to R848 are temporary and can be overcome with appropriate dosing kinetics (Fig. 3a, b). These findings combined indicate that R848 is tolerable, especially when delivered on a consistent repeated dosing schedule.

### R848 decreases cardiac and lean mass catabolism from PDAC-associated cachexia

**Fig. 4:**
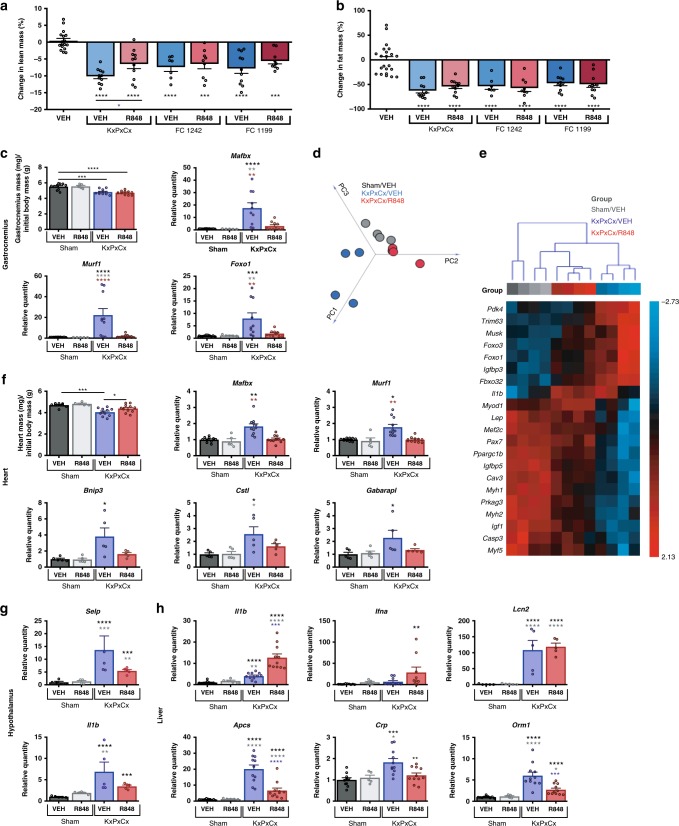
R848 improves molecular physiology markers related to cachexia. **a** Lean mass changes assessed by nuclear magnetic resonance (NMR) relaxometry, comparing absolute lean mass before implantation with tumor-free lean mass at end of study. Cell lines include KxPxCx, FC1199, and FC1242. **b** Fat mass changes assessed by NMR, comparing absolute quantity of fat mass prior to implantation with absolute quantity of fat mass at end of study. Statistics depict significance as compared with sham-operated vehicle-treated mice unless otherwise designated, via one-way ANOVA with Sidak multiple comparison correction. **c** Normalized gastrocnemius mass and gene expression of key catabolic transcripts for sham or KxPxCx-implanted animals treated with vehicle (VEH) or R848. **d** Principle component analysis of all 84 myopathy and myogenesis related transcripts in sham vehicle, and KxPxCx allocated to vehicle or R848. **e** Unsupervised clustering analysis of all myogenesis and myopathy related transcripts with a fold regulation >2 and adjusted *P* < 0.05 between groups. Expression values are normalized by SD for each transcript to depict variance from the mean. **f** Normalized heart mass and cardiac gene expression of catabolic and autophagy transcripts for sham or KxPxCx-implanted animals treated with vehicle (VEH) or R848. Gene expression for hypothalamus (**g**) and liver (**h**) are shown for sham-operated and KxPxCx-implanted mice treated with VEH or R848. Data are depicted as mean ± SEM, with individual data points shown. *P < 0.05; **P < 0.01; ***P < 0.001; ****P < 0.0001, with the color of symbol matched to the relevant comparison group: black asterisk, vs. sham-VEH; grey asterisk, vs. sham-R848; blue asterisk, vs. KxPxCx-VEH; red asterisk, vs. KxPxCx-R848

To assess the overall effects of tumor burden and R848 on body composition, lean mass and adiposity were measured using nuclear magnetic relaxometry immediately before tumor implantation and again before necropsy. Across all cell lines, tumor burden resulted in lean and adipose tissue wasting (Fig. 4a, b). For KxPxCx, R848-treated mice exhibited improvements in lean mass retention over vehicle-treated mice (*P* < 0.05). No improvements were noted in fat mass retention in any cell line tested.

At necropsy, R848 did not result in muscle catabolism in sham-operated animals, but did not rescue muscle loss resulting from KxPxCx engraftment (Fig. 4c). Nevertheless, skeletal muscle catabolic gene signatures were distinctly improved by treatment with R848. Compared with vehicle-treated KxPxCx-engrafted mice, R848-treated animals demonstrated decreases in the E3 ubiquitin ligases *Mafbx* and *Murf1*, as well as the transcription factor *Foxo1*. Targeted array profiling of skeletal muscle was performed for 84 transcripts related to myogenesis and myopathy, comparing sham-operated animals to KxPxCx-bearing animals with and without R848 (Supplementary Fig. 5).

Principle component analysis revealed that R848 modified the skeletal muscle transcriptome to become more similar to sham-operated animals than vehicle-treated tumor-bearing counterparts (Fig. 4d). Of these transcripts, 21 were found to differ significantly at a threshold of *α* = 0.05 with a fold change > 2.0 vs. one or more experimental groups (Fig. 4e). Skeletal muscle gene transcription more closely resembled sham-operated animals following R848, with the exception of the pro-inflammatory cytokine *Il1b* and muscle differentiation and repair transcription factor *Myod1*, which were upregulated in R848-treated KxPxCx-engrafted mice compared with vehicle-treated KxPxCx-engrafted mice and healthy controls.

In addition to improvements in the molecular physiology of skeletal muscle, KxPxCx-bearing animals treated with R848 demonstrated preserved heart mass (*P* < 0.05 vs. vehicle-treated KxPxCx, n.s. vs. sham). Quantitative reverse-transcriptase PCR (qRT-PCR) of cardiac muscle demonstrated that R848 decreased expression of *Mafbx* and *Murf1*, and autophagy-associated transcripts frequently upregulated in cardiac tissue during cachexia (*Bnip3*, *Ctsl*, *Gabarapl*) (Fig. 4f).

### Hypothalamic and systemic gene expression during PDAC are modified by R848

Cancer cachexia is commonly understood to result from pathogenic crosstalk between the tumor and a variety of physiological systems. With successful reduction in tumor burden, abnormal inflammatory signaling in peripheral tissues could improve due to loss of tumor-derived mediators associated with cachexia. Because of the improvements noted in appetite and behavior, the hypothalamus was queried for changes in inflammatory transcripts associated with cachexia. Consistent with prior studies, KxPxCx allograft-induced cachexia resulted in hypothalamic inflammation in vehicle-treated animals, with increases in expression of *Selp*, *Il1b*, *Il1r1*, *Tlr7*, *Ccl2*, and *Icam1* (Fig. 4g and Supplementary Fig. 6A). Treatment with R848 resulted in decreased hypothalamic inflammatory gene expression in a subset of these transcripts, including *Il1b* and *Selp*, but these changes did not approach statistical significance.

Given the liver’s central role in processing inflammatory stimuli, we hypothesized that R848 and tumor burden would each exert influences on hepatic gene expression. Consistent with previous studies, we found that tumor burden resulted in elevated hepatic *Il1b*, *Ifna*, *Lcn2*, *Apcs*, *Crp*, and *Orm1* (Fig. 4h). No changes were observed in these transcripts when R848 was delivered to healthy sham-operated animals. However, livers from KxPxCx animals treated with R848 had a distinct inflammatory profile from other experimental groups. Two transcripts, the cytokines *Il1b* and *Ifna*, were more elevated in tumor-bearing animals treated with R848 as compared with vehicle alone (*P* < 0.0001 and *P* < 0.01, respectively, analysis of variance (ANOVA) with Tukey’s test). One transcript, the acute stress response signal *Lcn2*, was equally increased in R848 and vehicle-treated tumor-bearing animals (*P* < 0.0001 vs. sham-operated vehicle for both groups). However, three classical acute phase reactants, *Apcs, Orm1*, and *Crp*, were significantly decreased in tumor-bearing animals treated with R848 compared with vehicle, consistent with decreased tumor burden-associated inflammatory responses.

As another means of assessing systemic immune responses during tumor progression and R848 therapy, the spleen was investigated for gross morphology, histology, and cell composition. Splenic mass at necropsy was markedly increased both due to KPC tumor burden and R848 treatment (Supplementary Fig. 6B). Histological analysis of spleens from R848-treated tumor-bearing animals revealed expansion of the white pulp, compatible with reactive lymphoid hyperplasia (Supplementary Fig. 6C). Flow cytometry performed on spleens from R848-treated KxPxCx-engrafted animals demonstrated, compared with vehicle-treated KxPxCx animals, an increase in CD11b^−^CD11c^+^ dendritic cells (*P* < 0.0001), a decrease in monocytes (*P* < 0.01), and an increase in CD3^+^CD4^+^ T cells (*P* < 0.05) (Supplementary Fig. 6D), suggestive of increased trafficking of dendritic cells to lymphoid tissue following R848.

### Continuous R848 treatment extends survival in murine PDAC

**Fig. 5:**
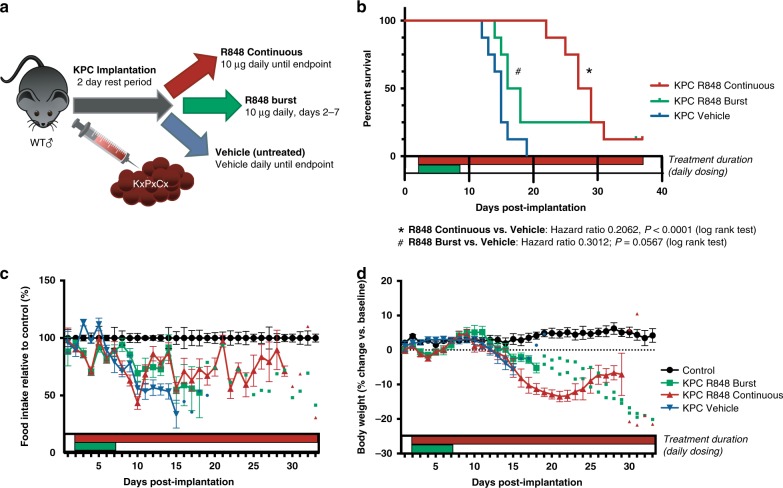
Continuous R848 monotherapy extends survival in allografted PDAC. Experimental design (**a**), Kaplan-Meier curve comparing sham-treated, burst-treated R848, and continuous-treated R848 KxPxCx-engrafted mice compared by log rank test (**b**), food intake relative to sham controls (**c**), and body weight relative to initial measurement (**d**). n = 8/group. Values are depicted as mean ± SEM, unless fewer than three subjects were remaining in a group, for which individual values are plotted

As R848 improved tumor burden and cachexia, we next investigated whether this translated to changes in survival and how treatment duration affected these outcomes. We therefore included two R848 treatment arms for survival analysis: a five-dose “burst” dosing regimen and a continuous dosing regimen (Fig. 5a). Animals were compared for duration of survival while being tracked for cachexia. Continuous treatment with R848 resulted in a near doubling of survival duration in KxPxCx-engrafted animals and 12.5% survival at 35 days (median survival 15d vehicle vs. 28d continuous-treated R848, Log-rank test *p* < 0.0001) (Fig. 5b). In contrast, burst treatment with R848 modestly extended survival, but had a sustained response in only a small proportion of subjects (median survival 15d vehicle vs. 17d burst-treated R848, Log-rank test *P* = 0.056). Although mice remained hypophagic despite R848 therapy in later stages of illness, food intake was improved relative to vehicle-treated KxPxCx-bearing animals through most of the study (Fig. 5c). Body weight continued to decrease over the course of the study among R848-continuous-treated mice, indicative of possible drug toxicity or the result of ongoing hypophagia (Fig. 5d). In addition to prolonged survival, mice treated with R848 exhibited normalized behavior for weeks following initiation of therapy, including decreased physical signs of illness (Supplementary Movie 1).

### Anti-tumor effects of R848 are mediated by host, not neoplastic, TLR7

**Fig. 6:**
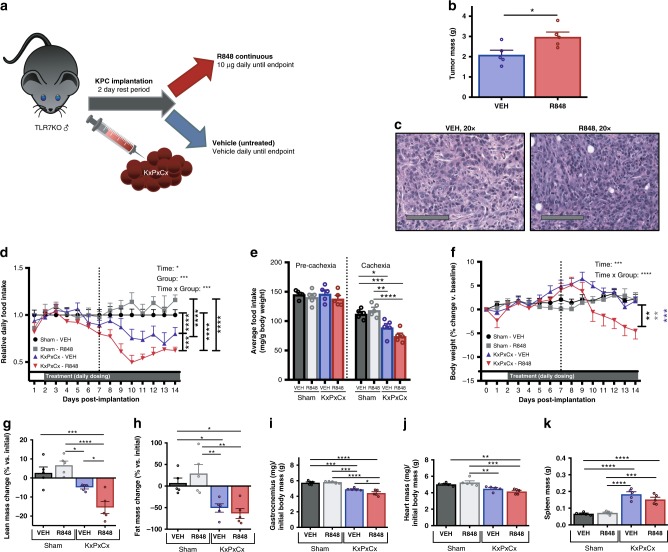
The anti-tumor and cachexia attenuating effects of R848 require stromal TLR7. Male TLR7 hemizygous null (TLR7KO) mice were implanted with TLR7 intact KxPxCx, then randomized to R848 or vehicle as previously described (**a**). Tumor size (**b**) was increased following R848 in this context, with representative histology shown in (**d**). Food intake (plotted as a function of time in **d** and as cumulative averages in **e**) and body weight (**f**) were both more severely affected during cachexia stage R848 treatment when TLR7 was absent in host tissues but present in neoplastic cells. This was accompanied by lean mass loss (**g**), no further changes in adipose wasting (**h**), skeletal muscle catabolism (**i**), cardiac muscle catabolism (**j**), and lack of splenic response to treatment (**k**). Statistical analyses were performed using two-way ANOVA during the cachexia stage to test for main effects of tumor and treatment, and the interaction thereof, with multiple-comparisons tests for main column effects. Values represent mean ± SEM. *P < 0.05; **P < 0.01; ***P < 0.001; ****P < 0.0001

The effects of TLR agonists on tumor immunity could be attributed to actions on neoplastic cells, stromal elements, host, or a combination thereof. Althoughour earlier results indicated that epithelial factors mediate R848 response, the changes in immune complexity of treated tumors led us to hypothesize that R848-induced tumor immunity also requires stromal TLR7. As TLR7 is an X-linked gene, male mice harboring hemizygous null deletions in TLR7 (TLR7KO) were used to query the role of host TLR7 in R848-mediated tumor response. TLR7KO mice were implanted with R848-responsive KxPxCx tumor cells and tracked for tumor growth and cachexia (Fig. 6a). Contrary to the results in TLR7-sufficient mice, KxPxCx-engrafted TLR7KO mice treated with R848 experienced increased tumor growth relative to vehicle-treated mice (Fig. 6b, c).

As TLR8 is not imidazoquinoline-sensitive in mice, we anticipated no R848 effect on host in TLR7KO mice. Indeed, although there was no decrease in food intake or body weight associated with treatment induction, due to these adverse effects being on-target and mediated exclusively through TLR7 in mouse, treatment groups began to diverge significantly during the cachexia stage. KxPxCx-engrafted TLR7KO animals treated with R848 developed significantly worse anorexia and weight loss compared with vehicle-treated counterparts (Fig. 6d–f). Simultaneously, KPC-bearing TLR7KO animals treated with R848 had exacerbated lean mass loss (Fig. 6g), skeletal muscle catabolism (Fig. 6i), and cardiac atrophy (Fig. 6j). Combined, these results confirm that host rather than neoplastic TLR7 is necessary for R848’s beneficial effects and substantiate caution that TLR7 activity may increase tumor burden if unchecked by immune response.

### Tlr7 is commonly expressed in the stroma in human pancreatic neoplasms

**Fig. 7:**
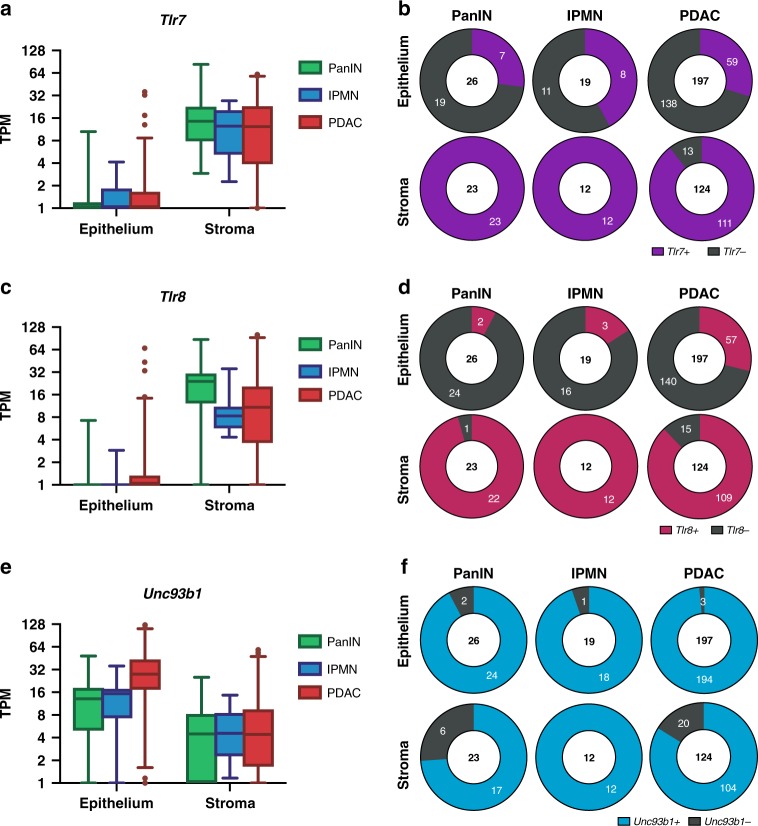
*Tlr7* is common in the stroma but not in the epithelium of human pancreatic neoplasms. Left: Transcript count per million (TPM) from RNA-seq in epithelial and stromal laser capture microdissected samples from PanIN, IPMN, and PDAC samples for *Tlr7* (**a**), *Tlr8* (**c**), and *Unc93b1* (**e**). Data displayed as interquartile range, with whiskers depicting 2.5th–97.5th perecentile. Right: Proportions of samples positive and negative for *Tlr7* (**b**), *Tlr8* (**d**), and *Unc93b1* (**f**) based on the threshold of 1 TPM. Sample size for each category of neoplasm (PanIN, IPMN, and PDAC) and tumor compartment (epithelium and stroma) are shown in the center of the plot, with the outer values depicting absolute frequency of positive and negative expression

Based on the differential effects we observed depending on whether TLR7 was present in tumor stroma, we investigated the frequency of R848-responsive genes in tumor compartments using an RNA-seq library of laser-capture microdissected human pancreatic lesions. To determine whether expression differed over the course of disease development, we queried two types of precursor lesions, pancreatic intraepithelial neoplasia (PanIN) and intraductal papillary mucinous neoplasia (IPMN), and PDAC. The vast majority of samples expressed stromal *Tlr7*, including 12/12 IPMN samples, 23/23 PanIN samples, and 111/124 PDAC samples (Fig. 7a). *Tlr7* was far less abundant in epithelial samples, with at least 1 TPM in 8/19 IPMN samples, 7/26 PanIN samples, and 59/197 PDAC samples (Fig. 7b). Although murine TLR8 is generally considered inactive, human TLR8 is abundantly expressed and responsive to ligands including ssRNAs and imidazoquinolines. We found *Tlr8* demonstrated a gene expression distribution similar to *Tlr7* across stages of pancreatic neoplasia, with a majority of stromal samples expressing at least one TPM and only a minority of epithelial samples demonstrating positive *Tlr8* expression (Fig. 7c, d). The transcript encoding the obligate intracellular shuttle protein for TLR7, uncoordinated homolog 93 B1 (*Unc93b1)*, was typically present in both epithelium and in the stroma, indicating the machinery to traffic TLR7 to its active signaling compartment was intact (Fig. 7e, f). These data indicate that most patients with pancreatic neoplasms have tumor gene expression profiles compatible with therapeutic responses to TLR7 agonists.

## Discussion

**Fig. 8:**
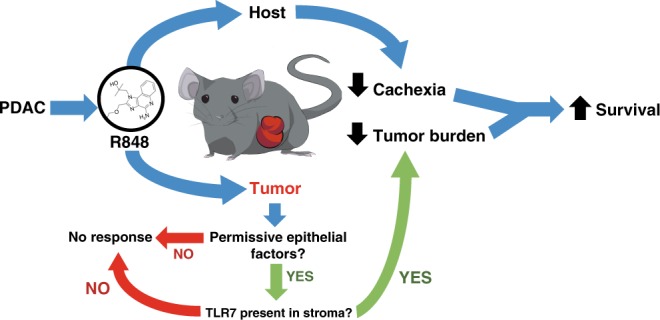
Graphical summary of findings. We find that in murine models of PDAC, R848 acts on both host and tumor to promote beneficial survival responses. In host, we observe decreased manifestations of cachexia by behavioral and molecular metrics. In tumor, we observe that dependent on whether epithelial cells recruit R848-sensitive stroma, TLR7 stimulation mediates anti-tumor responses including increased T-cell infiltration and cytotoxic activity. These factors converge to extend survival in orthotopic murine models of PDAC

In this study, we demonstrate that R848 remodels the tumor immune microenvironment and host responses in PDAC, and is ultimately associated with improved survival (Fig. 8). The anti-tumor response elicited by R848 is characterized by the formation of tertiary lymphoid structures, increased CD8^+^ T-cell infiltration with evidence of increased activation and cytotoxicity, and decreased Treg concentration, all of which are associated with improved prognosis in human malignancy^23–25^. Based on the lack of tumor reduction and exacerbation of cachexia in TLR7-deficient hosts, we report that the beneficial effects of R848 require host rather than neoplastic TLR7. Given that not all epithelial PDAC tumor cell lines demonstrate tumor response to R848, we show that R848 sensitivity is additionally dependent on neoplastic cell intrinsic factors. This is consistent with recent work illustrating that tumor cell intrinsic factors shape the tumor immune microenvironment, including the extent of T-cell infiltration and the ability to respond to combination immunotherapy during PDAC^26^. Although TLR7 agonists are classically associated with acute illness responses, we observed that ongoing therapy resulted in tachyphylaxis of sickness responses without diminution of therapeutic effect. The anti-tumor response coupled with the lack of persistent sickness resulted in improvements in cachexia and decreases in tissue catabolism during the course of treatment. This represents a key difference from many cytotoxic chemotherapies and suggests some classes of immunotherapy may be beneficial in the treatment of cachexia-associated malignancies.

A growing array of immunotherapy modalities show considerable promise, such as tumor vaccines, cytokines, monoclonal antibodies, and immunostimulatory small molecules, yet crucial unknowns persist in their clinical application^27,28^. Immune-related adverse events with T-cell checkpoint inhibitors are common^29^. Immunotherapy optimization will likely become even more complex as other clinical variables such as cachexia are considered. Options for PDAC patients with cachexia often prove startlingly narrow, with even the most successful regimens such as FOLFIRINOX demonstrating decreased therapeutic efficacy in patients with tissue wasting^30,31^. Cachexia increases complications during cancer treatment, decreases therapeutic tolerance, and is a key determinant of survival duration in PDAC clinical trials^31–33^. However, with further optimization, it is possible that immune-based therapies could provide effective tumor responses without the same degree of systemic toxicity as cytotoxic chemotherapy.

Several recent studies highlight the dual potential for benefit and harm of TLR7 agonists, which hold substantial promise as anti-tumor agents but cause acute toxicities^34–36^. Indeed, we observed that treatment induction was associated with acute hypophagia, weight loss, and decreased locomotor activity. However, these effects quickly desensitize, consistent with the tightly regulated role of TLR7 activity in immunity. Of note, recent work demonstrated that the TLR7/8/9 antagonist IMO-8503 improved lean mass retention and decreased muscle catabolism in the Lewis lung carcinoma (LLC) cachexia model, without any changes to tumor size^37^. Based on this finding, we speculate that the protective effect we observe in muscle is due to desensitization to pathogenic TLR7 signaling at the level of host tissues via therapeutic induction of immune tolerance. However, it is also possible that tumor-intrinsic factors mediate the decrease in cachexia. Unlike in the case of TLR7/8/9 antagonism in LLC, R848 decreases tumor size in most PDAC cell lines tested. However, based on how FC1199-derived tumors did not respond to R848, yet animals still had improvements in cachexia, it is likely that R848 confers tumor-extrinsic protective signaling in host tissues.

TLRs are components of the innate immune system that respond to common molecular motifs in pathogens^38^. Both TLR7 and TLR8 are endosomal receptors that detect a variety of purine-rich ssRNA species^8^. In mouse, TLR7 is predominantly expressed in plasmacytoid dendritic cells and B cells, with context-dependent expression in subsets of macrophages and T cells^39^. Following inflammatory stimuli, TLR7 is ubiquitinated and packaged into COPII-coated vesicles by the endoplasmic reticulum (ER)-resident chaperone UNC93B1^40,41^. Upon binding ssRNA, TLR7 signaling results in the production of pro-inflammatory cytokines and type I interferon, T-cell proliferation, and induction of T_H_1 responses^42,43^. We observed many of these elements within PDAC tumors treated with R848, including CD8^+^ T-cell proliferation and effector function, and decreased T_H_2 polarization among CD4^+^ T cells. However, the sweeping changes to immune populations with R848 that TLR7 agonists implies widespread effects on numerous immune cell lineages during malignancy.

A cardinal feature of cancer is the evasion of immunity via immunosuppressive signaling within the tumor microenvironment^44^. This feature is common in PDAC: both local immunosuppression and structural barriers, such as stromal desmoplasia, are key therapeutic challenges. The TLR7 agonist imiquimod is FDA-approved as a monotherapy for basal cell carcinoma and the potential for TLR agonists is expanding into other malignancies^9^. In some cases, TLR7 stimulation of T cells alone is sufficient for anti-tumor responses: nanoparticle delivery of R848 to CD8^+^ T cells results in increased anti-tumor immunity and prolonged survival in a murine colorectal cancer model^20^. TLR7 agonists also demonstrate benefit in combination with doxorubicin in T-cell lymphoma^19^, with vaccination in bladder cancer^18^, and with radiotherapy in gastrointestinal tumors^45^.

Although this study did not employ a combinatorial approach, there is strong rationale for future studies investigating efficacy and tolerability of TLR7/8 agonists as part of combination therapy. On monotherapy alone, KPC-derived mice treated with R848 eventually developed tumor progression leading to cachexia and mortality. Interestingly, adverse responses to R848 such as hypophagia and weight loss demonstrated relatively rapid desensitization kinetics, but anti-tumor responses were substantially more robust with long-term dosing. This suggests that R848’s adverse systemic effects and anti-tumor responses are likely mediated by different cell types with functionally distinct signaling kinetics and receptor modulation. Similar uncoupling of central nervous system immune tolerance and anti-tumor responses is observed with tumor necrosis factor, suggesting that this phenomenon may occur in a variety of contexts^46^. Indeed, most pathogen-associated molecular patterns and damage-associated molecular patterns demonstrate behavioral tachyphylaxis following repeated stimuli, similar to the R848 initial illness response^17,47^. In contrast, the limited course of illness responses is not observed with cytotoxic chemotherapy, which induces dose-dependent deleterious effects^48^, nor checkpoint inhibitors, which are associated with acute and chronic immune-related adverse events^29^. It therefore remains possible that with TLR7/8 agonists, lower doses or shorter courses of other therapies can be used to attain effective tumor response without exacerbating cachexia.

Further studies will be crucial as this class of drug is evaluated for clinical use. We used syngeneic OT models of pancreatic cancer to avoid the risk of global pancreatic dysfunction, which can occur in genetically engineered mouse models of pancreatic cancer and confounds assessment of cachexia. As genetically engineered mouse models provide a more accurate recapitulation of tumor biology, future work should utilize these models to evaluate TLR agonists. In addition, it will be beneficial to evaluate other aspects of cachexia, including muscle strength, motor performance, stamina, as well as cognitive and affective components of behavior.

Several factors are important to consider as part of the successful translation of these findings into clinical use. The foremost of these is that TLR8 confers different ligand specificity in mice vs. humans. Although imidazoquinolines are capable of stimulating both human TLR7 and TLR8, they do not bind to murine TLR8 and therefore only result in TLR7-dependent transduction in mouse^49,50^. R848 was used in these studies, because it is substantially more potent than the TLR7-specific imiquimod^8^. However, before clinical use, R848 effects on tumor-intrinsic and tumor-extrinsic biology should be further characterized in a model system that expresses TLR8. Alternatively, a potent TLR7-specific agonist could be used for translation of these studies, such as CL097 or 852A^51^. Prior clinical trials using systemic TLR7 agonists were associated with toxicities including fever and fatigue^52^; however, our data demonstrate that these effects are acute rather than chronic. We recognize that drugs that cause acute toxicity present unique challenges in human trials and clinical use. However, agents with more severe toxicities than described here are ubiquitous in clinical oncology. Indeed, many chemotherapies are associated not only with anorexia and weight loss, but also pose major risks to the heart, bone marrow, lungs, nerves, kidneys, gastrointestinal tract, and more^53–57^. However, these risks are accepted due to the morbidity and mortality associated with cancer progression. Although it is unknown whether systemic dosing paradigms will be tolerable in patients with pancreatic cancer, existing clinical data on topical imiquimod and our mouse studies on systemic R848 are encouraging for long-term use of this drug class.

Finally, these results highlight the importance of evaluating immunotherapy effects using a whole animal physiology approach. Outcomes from PDAC can be thought of as the sum of the two domains: the tumor itself and the host in which the disease takes place. At the same level of tumor burden, a more resilient host will have improved outcomes relative to a weakened host, as illustrated by the increased morbidity and mortality seen in patients with cachexia. The most effective treatment paradigms for PDAC should be effective against the primary tumor and serve neutral or beneficial roles systemically. Although not all forms of immunotherapy will necessarily have positive effects on host pathologies such as cachexia, agents such as R848 that confer local and systemic benefits will be highly valuable as we strive to improve outcomes in pancreatic cancer.

## Methods

### Experimental design

The primary objective of this study was to simultaneously evaluate tumor and cachexia responses to the immunotherapy R848 in preclinical models of PDAC. Furthermore, we aimed to uncover mechanistic data regarding the necessary components to observe anti-tumor immunity, including testing for the effects of neoplastic heterogeneity and stromal immune receptor expression. Finally, we sought to verify whether the mechanistic factors required for anti-tumor response were present in a large set of patient pancreatic neoplasia samples. To assess cachexia outcomes and tumor response, we used a murine PDAC-associated cachexia model recently developed in our laboratory^58^. Three distinct neoplastic cell lines were used to account for neoplastic heterogeneity in drug response. TLR7 knockout mice were used to query the role of host biology in R848 response. Human data were used to perform clinical correlation for the gene expression pattern we found is required for favorable R848 response. Sample sizes were selected based on pilot data assessing tumor mass at the endpoint and anorexia during cachexia stage. Blinding was performed for any qualitative analysis, including histology interpretation. Endpoints and data collection were designated a priori as described in subsections below.

### Animals

Male C57BL6/J mice (JAX, catalog number #000664) and TLR7^−/y^ mice backcrossed to the C57BL6/J background (JAX, catalog number #008380) were maintained in standard housing at 26 °C and 12 h light/12 h dark cycles. Animals were provided ad libitum access to water and food (Rodent Diet 5001; Purina Mills). In the week prior to implantation, animals were transitioned to individual housing to acclimate to experimental conditions. Animal food intake and body weight were monitored daily at 0900–1000 h. Treatment was delivered from 1200–1300 h. All studies were performed in accordance with the NIH Guide for the Care and Use of Laboratory Animals, with all protocols receiving ethical evaluation and study approval by the Oregon Health and Science University Institutional Animal Care and Use Committee (IACUC).

### KPC-derived neoplastic cell lines

The KPC model expresses double heterozygous knock-in pancreas-specific conditional alleles KRAS^G12D^ and TP53^R172H^ via the PDX-1-Cre driver, and is a preferred model of PDAC for its close recapitulation of human disease. To study KPC-derived tumors representing a spectrum of neoplastic cell heterogeneity, we used three epithelial tumor cell lines each derived from a distinct KPC mouse in a syngeneic C57BL6 background. These were provided generously by Dr. Elizabeth Jaffee (KxPxCx) and Dr. David Tuveson (FC1242 and FC1199)^59,60^. All three are male cell lines and all express *Tlr7* (Supplementary Fig. 7 and Source Data File). Cells were maintained in RPMI 1640 supplemented with 10% fetal bovine serum, 1% minimum essential medium non-essential amino acids, 1 mM sodium pyruvate, and 50 U/mL penicillin/streptomycin (Gibco), with incubators maintained at 37 °C and 5% CO_2_.

### Generation of PDAC model and treatment paradigm

Eight-week-old male C57BL/6J wild type or TLR7^−^ mice were orthotopically or IP implanted with 10^6^ KPC-derived neoplastic cells or equivalent volume saline, then randomized 2 days later to daily IP R848 (10 µg) or vehicle until endpoint. R848 was obtained from Enzo Life Sciences (#NC9739083). In studies comparing cachexia status and tumor immune response, all animals were killed when any study arm developed end-stage cachexia or tumor burden exceeding IACUC guidelines. End-stage cachexia was defined based on food intake rather than body weight. This is because it is difficult to quantify lean mass changes in a living subject in this model, as loss of host tissues develops simultaneously with growth of tumor mass. We therefore employed a combination of quantitative metrics (persistent absolute daily food intake of <1.5 g), and qualitative metrics (physical exam findings including hunching or evidence of respiratory insufficiency) to assess cachexia status. For studies comparing tumor and cachexia responses cross-sectionally, all groups were killed when the majority of subjects in any group demonstrated evidence of end-stage cachexia. In studies comparing survival duration, individual subjects were killed following onset of IACUC-designated signs of moribund behavior.

### Tumor fixation and histology

Tumor samples were fixed in formalin and paraffin embedded, then sectioned for further analysis. Hematoxylin and eosin stains were evaluated for tumor grade and immune cell characteristics by a board-certified pathologist blinded to experimental group (TKM).

### Flow cytometry

Following killing, tumor-bearing mice were perfused with phosphate-buffered saline to clear tissue of vascular immune cells. Tumors were collected in Dulbecco’s modified Eagle’s medium (DMEM) on ice, then minced and enzymatically digested in DMEM containing 1.0 mg/mL Collagenase IV (Gibco, #17104–019), 1.0 mg/mL Soybean Trypsin Inhibitor (Gibco; #17075–029), and 50 U/mL DNAse 1 (Roche; #10104159001). Samples were incubated for 30 min at 37 °C with agitation at 125 r.p.m., filtered through 100 µM cell strainers, centrifuged at 400 × *g* for 5 min at 4 °C, then resuspended and immediately stained for flow cytometry. Antibodies and reagents to label cells can be found in Supplementary Table 1. Flow cytometry was performed on a BD Fortessa and data were analyzed in FlowJo software.

### Multiplex immunohistochemistry staining

Representative tumors from vehicle- and R848-treated KxPxCx-implanted animals were stained using a 23-marker mIHC panel, as previously described^21^. Briefly, tumors were fixed in paraformaldehyde, paraffin-embedded, and sectioned immediately prior to staining. Antibodies used for sequential staining are described in Supplementary Table 2. Histofine Simple Stain Max Reagents for anti-rat (Nichirei Biosciences, #414311 F) and anti-rabbit (#414144 F) were used for detection of primary antibodies, and the final stain development performed with AEC (Vector Lab, SK-4200). Slides were coverslipped in water and scanned following each marker using a Leica Aperio. Hematoxylin stains were performed at cycle 2 and after the final cycle to identify cells and account for any tissue loss.

### Analysis of multiplex immunohistochemistry

ImageScope software (Leica) was used to select five ROIs of fixed size (5000 × 5000 pixels) per sample, collectively representing a minimum of 50% of the tumor cross-sectional area. On average, ROIs contained 47,476 cells (range: 28,236–61,573), with an average of 237,380 cells analyzed per tumor (range: 165,960–266,659). ROIs were registered for all markers using the SURF algorithm in MATLAB. Registered regions were imported into FIJI-ImageJ to extract AEC signal from background with color deconvolution and generate single cell segmentation masks, upon which merged pseudocolor images were generated for markers of interest. Cell Profiler was used to measure the mean intensity of each marker for each segmented cell. The output was imported into FCS Express 6.0 Image Cytometry and manually gated with picture plots to visually validate the expression of each cell as true positive or true negative for each marker through the gating hierarchy. The hierarchical gating strategy for identification of cell types and functional markers is described in Supplementary Fig. 1B. Results were exported into SPSS (IBM) and Orange (University of Ljubljana) to perform unsupervised clustering analysis, and to generate heat maps depicting cell identities and activation states. Statistical analysis was performed using Prism 8.0 software, with per-ROI or per-tumor analysis as specified.

### Analysis of cachexia and tumor outcomes

Animal food intake and body weight were monitored daily, with bedding sieves to account for food spillage. Body temperature and voluntary locomotor activity were measured via implanted Minimitter tracking devices on 5 min recording intervals (MiniMitter, Bend, OR, USA). Body composition was assessed at the designated time points using EchoMRI nuclear magnetic resonance relaxometry. Necropsies were performed by observers blinded to the treatment group to obtain mass measurements of tumor, heart, gastrocnemius, and spleen. All discrete tumor masses were included in the weight measurement. Tumor, hypothalamus, heart, gastrocnemius, and liver were immediately stored in RNAlater for gene expression analysis (Ambion).

### Gene expression assays

Following tissue homogenization, RNA was purified with the RNeasy Mini Kit (Qiagen), then reverse transcribed with the High Capacity cDNA Reverse Transcription Kit (Life Technologies). qRT-PCR was performed using TaqMan reagents and the primer probes listed in Supplementary Table 3, with normalization to 18S using the ddCT method. Gene expression analysis was performed on gastrocnemius with the Myogenesis and Myopathy RT2 Profiler Array (#PAMM-099Z, Qiagen). Log_2_-transformed expression values were imported into Orange, normalized to SDs from the mean for each transcript, and subjected to principal component analysis (PCA) and unsupervised clustering analysis.

### RNA-seq of stromal and epithelial compartments in human pancreatic neoplasia

To compare gene expression in stromal and epithelial samples for key R848 response elements in human neoplasia, we queried a dataset of RNA-seq using human cohorts of PanIN, IPMN, and PDAC, as previously described.^61^ Samples were normalized to transcripts per million for analysis and were stratified for level of expression and overall positive rate for the transcripts *Tlr7*, *Tlr8*, and *Unc93b1*.

### Statistics

Prism 8.0 software was used for statistical analyses. For cross-sectional analyses of two groups, we performed *T*-tests,and when greater than two groups, ANOVA with Tukey’s test for multiple comparisons between all experimental groups or Sidak’s test for comparisons against a single control group. For longitudinal comparisons of cachexia parameters, data were grouped into the bins of pre-cachexia and cachexia. Differences for these variables are only expected between groups during the cachexia stage, defined as persistently decreased food intake of 10% or more relative to control subjects. During cachexia stage, we performed two-way ANOVA for the main effects of experimental group and time, the interaction thereof, and Tukey’s test for multiple comparisons of the main column effect. For interpretation of survival data, we did not expect the magnitude of effect to depend on duration of exposure nor did we expect the differences to emerge at relatively early time points, and therefore selected the Log-rank test for comparison of groups with respect to median survival and hazard ratio. All statistical tests were performed as two-tailed analyses.

### Reporting summary

Further information on research design is available in the Nature Research Reporting Summary linked to this article.

## Supplementary information

Supplementary Information

Description of Additional Supplementary Files

Supplementary Movie 1

Reporting Summary

Source Data

## Data Availability

All data associated with this study are available in the main text or the supplementary materials. Extended information associated with flow cytometry, quantitative multiplex IHC, RT2 Profiler array data, laser-capture microdissection RNA-seq, and cell lines are available as a Source Data file.
