# Publisher Correction: The TLR7/8 agonist R848 remodels tumor and host responses to promote survival in pancreatic cancer

**DOI:** 10.1038/s41467-019-13151-z

**Published:** 2019-11-15

**Authors:** Katherine A. Michaelis, Mason A. Norgard, Xinxia Zhu, Peter R. Levasseur, Shamilene Sivagnanam, Shannon M. Liudahl, Kevin G. Burfeind, Brennan Olson, Katherine R. Pelz, Diana M. Angeles Ramos, H. Carlo Maurer, Kenneth P. Olive, Lisa M. Coussens, Terry K. Morgan, Daniel L. Marks

**Affiliations:** 1 Medical Scientist Training Program, Oregon Health & Science University, Portland, OR USA; 2 Brenden-Colson Center for Pancreatic Care, Oregon Health & Science University, Portland, OR USA; 3 Papé Family Pediatric Research Institute, Oregon Health & Science University, Portland, OR USA; 4 Department of Computational Biology, Oregon Health & Science University, Portland, OR USA; 5 Department of Cell, Developmental and Cancer Biology, Knight Cancer Institute, Oregon Health & Science University, Portland, OR USA; 6 Departments of Medicine and Pathology and Cell Biology, Columbia University Medical Center, New York, NY USA; 7 Department of Pathology, Oregon Health & Science University, Portland, OR USA

**Keywords:** Cancer microenvironment, Pancreatic cancer, Immunotherapy, Feeding behaviour, Metabolic diseases

Correction to: *Nature Communications* 10.1038/s41467-019-12657-w, published online 15 October 2019.

The original HTML and PDF of this Article contained errors in Figs. 3 and 4. All panels for Figures 3 and 4 were inadvertently inverted. This has been corrected in both the HTML and PDF versions of the Article.

