# Supplementary material for: The TLR7/8 agonist R848 remodels tumor and host responses to promote survival in pancreatic cancer
Source: Nat Commun. 2019 Oct 15;10:4682. doi: 10.1038/s41467-019-12657-w (PMC6794326; doi:10.1038/s41467-019-12657-w)
Supplement: Supplementary file 1 — Supplementary Information [file 41467_2019_12657_MOESM1_ESM.pdf]

Manuscript Supplementary Information:

The TLR7/8 agonist R848 remodels tumor and host responses to promote survival in pancreatic cancer

Authors:
Katherine A. Michaelis, Mason A. Norgard, Xinxia Zhu, Peter R. Levasseur, Shamilene Sivagnanam, Shannon M. Liudahl, Kevin G. Burfeind, Brennan Olson, Katherine R. Pelz, Diana M. Angeles Ramos, H. Carlo Maurer, Kenneth P. Olive, Lisa M. Coussens, Terry K. Morgan, Daniel L. Marks

Supplementary Methods

Supplementary Table 1: Materials used for flow cytometry

Target and conjugate	Name	Manufacturer	Catalog	Dilution	FMO Prepared
CD11b FITC	Integrin alpha M	BioLegend	#101206	1:200	Yes
CD11c APC-Fluor780	Integrin, alpha X (complement component 3 receptor 4 subunit)	eBioscience	#47-0114-82	1:400	Yes
CD19 BV650	Cluster of differentiation 19	BioLegend	#115541	1:400	No
CD3 BV786	Cluster of differentiation 3	BD Horizon	#564010	1:200	Yes
CD4 BV605	T-cell surface glycoprotein CD4	BD Horizon	#563151	1:400	No
CD45 PE-Cy7	Protein tyrosine phosphatase, receptor type, C	BD Pharmingen	#552848	1:4000	No
CD8a BV711	Cluster of differentiation 8a	BD Horizon	#563046	1:200	No
F4/80 APC	EGF-like module-containing mucin-like hormone receptor-like 1	BioLegend	#123116	1:400	Yes
FOXP3 PE	Forkhead box P3	eBioscience	#12-5773-82	1:200	Yes
Ly6C PerCP	Lymphocyte antigen 6 complex, locus C1	BioLegend	#128028	1:400	Yes
Ly6G Alexa Fluor 700	Lymphocyte antigen 6 complex locus G6D	BioLegend	#127622	1:400	Yes
MHCII eFluor450	MHC class II antigen	eBioscience	#48-5321-82	1:1000	No
NK1.1 PE-Dazzle	Killer cell lectin-like receptor subfamily B, member 1	BioLegend	#108748	1:200	Yes
Live Dead Aqua	(N/A)	Invitrogen	#L34957	1:500	N/A

Supplementary Table 2: Antibodies used for mIHC

Target	Name	Manufacturer	Catalog	Dilution
B220	Protein tyrosine phosphatase receptor type C isoform B220	BD Biosciences	#550286	1:100
BTK	Bruton's tyrosine kinase	LS Bio	#LS-C180161	1:200
CD11b	Integrin alpha M	Abcam	#133357	1:3000
CD11c	Integrin, alpha X (complement component 3 receptor 4 subunit)	Cell Signaling	#97585	1:100
CD206	Cluster of Differentiation 206	Abcam	#64693	1:1000
CD3	Cluster of differentiation 3	Thermo	#RM-9107-S	1:300
CD4	T-cell surface glycoprotein CD4	Cell Signaling	#25229	1:100
CD45	Protein tyrosine phosphatase, receptor type, C	BD Biosciences	#550539	1:50
CD8	Cluster of differentiation 8	eBioscience	#14-0808-82	1:100
CD86	Cluster of Differentiation 86	eBioscience	#14-0862-81	1:100
CSF-1R	Colony stimulating factor 1 receptor	Santa Cruz	#Sc-692	1:250
EOMES	Eomesodermin	Abcam	#183991	1:1000
F4/80	EGF-like module-containing mucin-like hormone receptor-like 1	Serotec	#Cl:A3-1	1:200
FOXP3	Forkhead box P3	eBioscience	#14-5773-82	1:100
GATA3	GATA Binding Protein 3	Abcam	#199428	1:100
GZMB	Granzyme B	Abcam	#4059	1:200
Ki67	Marker of proliferation Ki-67	Abcam	#15580	1:5000
Ly6G	Lymphocyte antigen 6 complex locus G6D	eBioscience	#551459	1:200
PANCK	Cytokeratin	Abcam	#ab27988	1:100
PDL-1	Programmed death-ligand 1	Cell Signaling	#13684	1:50
RORyt	RAR-related orphan receptor gamma	Abcam	#ab207082	1:100
TCF1	Transcription factor T-cell factor 1	Cell Signaling	#2203s	1:100
TIM3	T-cell immunoglobulin and mucin-domain containing-3	Cell Signaling	#83882	1:200

Supplementary Table 3: Primer-probes used for qRT-PCR

Gene	Name	Catalog	Tissue(s)
Mafbx	Muscle atrophy F-box	Mm00399518_m1	Gastrocnemius, heart
Murf1	Muscle ring finger 1	Mm01185221_m1	Gastrocnemius, heart
Foxo1	Forkhead box O1	Mm00490672_m1	Gastrocnemius
Bnip3	BCL2 Interacting Protein 3	Mm01275600_g1	Heart
Ctsl1	Cathespin L1	Mm00515597_m1	Heart
Gabarapl	GABA Type A Receptor Associated Protein Like	Mm00457880_m1	Heart
Il1b	Interleukin-1 beta	Mm01336189_m1	Hypothalamus, liver
Il1r1	Interleukin 1 receptor type 1	Mm00434237_m1	Hypothalamus
Selp	P-selectin	Mm00441295_m1	Hypothalamus
Tlr7	Toll-like receptor 7	Mm00446590_m1	Hypothalamus
Ccl2	C-C motif chemokine ligand 2	Mm00441242_m1	Hypothalamus
Cxcl10	C-X-C motif chemokine 10	Mm00445235_m1	Hypothalamus
Icam1	Intercellular adhesion molecule 1	Mm00516023_m1	Hypothalamus
Apcs	Amyloid P component, serum	Mm00488099_g1	Liver
Crp	C-reactive protein	Mm00432680_g1	Liver
Orm1	Orosomucoid 1	Mm00435456_g1	Liver
Lcn2	Lipocalin 2	Mm01324470_m1	Liver
Ifna	Interferon alpha-1	Mm03030145_gH	Liver
18s	18s ribosomal subunit	4352930E	All

A

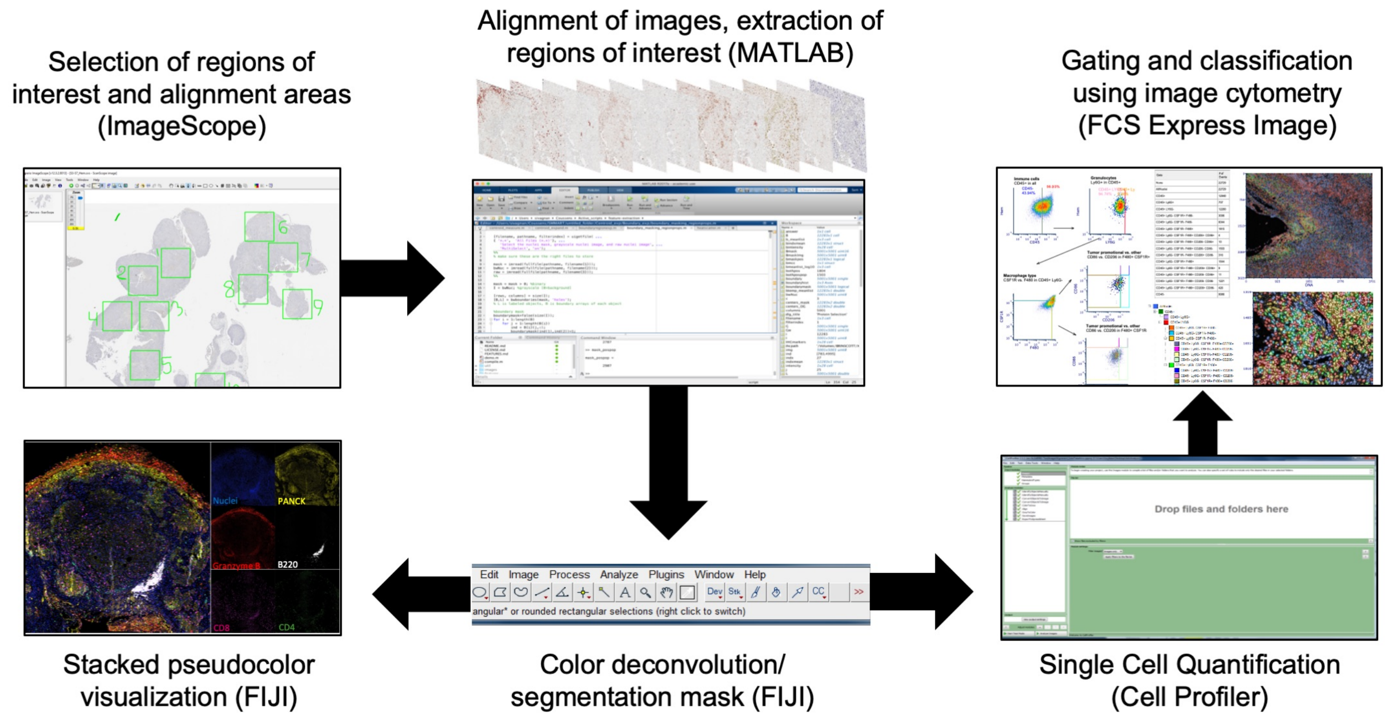

B

Type	Class	Identity	Subclass	Functional marker 1	Functional marker 2	Functional marker 3	Functional marker 4	Functional marker 5
Not Immune CD45-	Neoplastic PANCK+			PDL-1	Ki67			
	Fibroblasts/Other PANCK-			PDL-1	Ki67			
Immune CD45+	Lymphocytes	B Cells B220+		BTK	PDL-1	Ki67		
		CD4+ T Cell (CD3+ CD4+)	TH0/TH1 (Neg for all)	TIM3	TCF1	Granzyme B	Ki67	
			TH2 GATA3+	TIM3	TCF1	Granzyme B	Ki67	
			TH17 RORGT+	TIM3	TCF1	Granzyme B	Ki67	
			Treg FOXP3+	TIM3	TCF1	Granzyme B	Ki67	
		CD8+ T Cell	CD3+	EOMES	TIM3	TCF1	Granzyme B	Ki67
			CD3-	EOMES	TIM3	TCF1	Granzyme B	Ki67
		Other T Cell	CD4+ CD8+	EOMES	TIM3	TCF1	Granzyme B	Ki67
			CD4- CD8-	EOMES	TIM3	TCF1	Granzyme B	Ki67
	Myeloid cells CD11B+	Macrophages (F480+) CSF1R+ vs. CSF1R-	CD206+ / CD11C-	PDL-1	Ki67			
			CD206+ / CD11C+	PDL-1	Ki67			
			CD206- / CD11C-	PDL-1	Ki67			
			CD206- / CD11C+	PDL-1	Ki67			
		DCs (F480-)	CD206+ / CD11C+	PDL-1	Ki67			
			CD206- / CD11C+	PDL-1	Ki67			
		Granulocytes (Ly6G+)		PDL-1	Ki67			
		Other		PDL-1	Ki67			

Supplementary Figure 1. Workflow of quantitative multiplex immunohistochemistry analysis. A) Experimental pipeline of image acquisition, alignment, extraction of regions of interest, image assembly, cell quantitation, and image cytometry. B) Gating strategy used for image cytometry mIHC analyses.

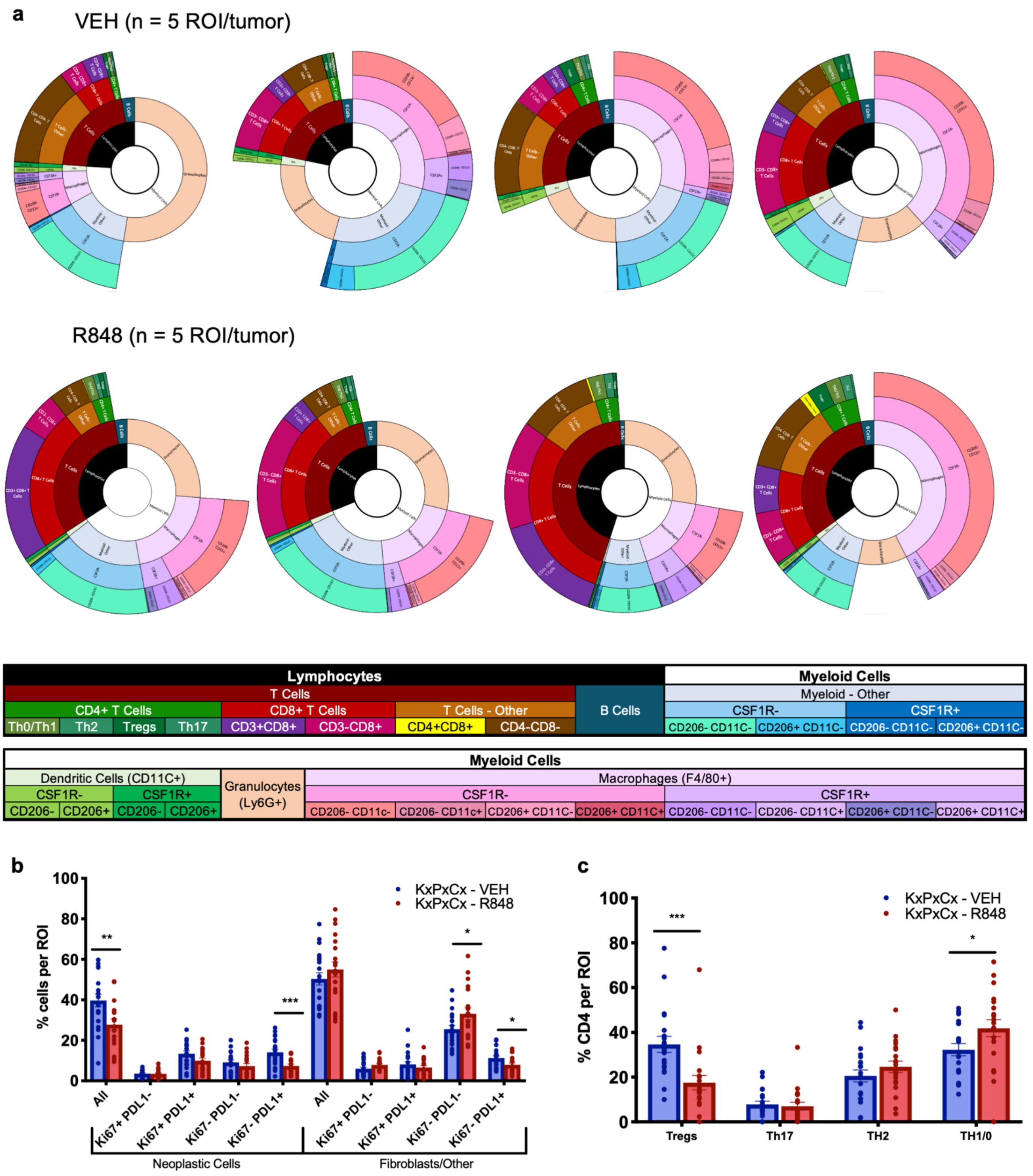

Supplementary Figure 2. Immune complexity mIHC analyses within KxPxCx-derived tumors following R848 treatment versus vehicle. A) Sunburst plots depicting the immune composition of each tumor within the dataset, derived from 5 ROIs representing greater than 50% of total section area. B) Proportion of proliferating (Ki67$^+$) and exhaustion-promoting (PDL1$^+$) cells in neoplastic and fibroblast/other compartments of the tumor, depicted as % per ROI within treatment groups vehicle and R848. C) Proportions of CD4 subsets within vehicle and R848-treated KxPxCx tumors. *, *P*<0.05; **, *P*<0.01; ***, *P*<0.001; ****, *P*<0.0001

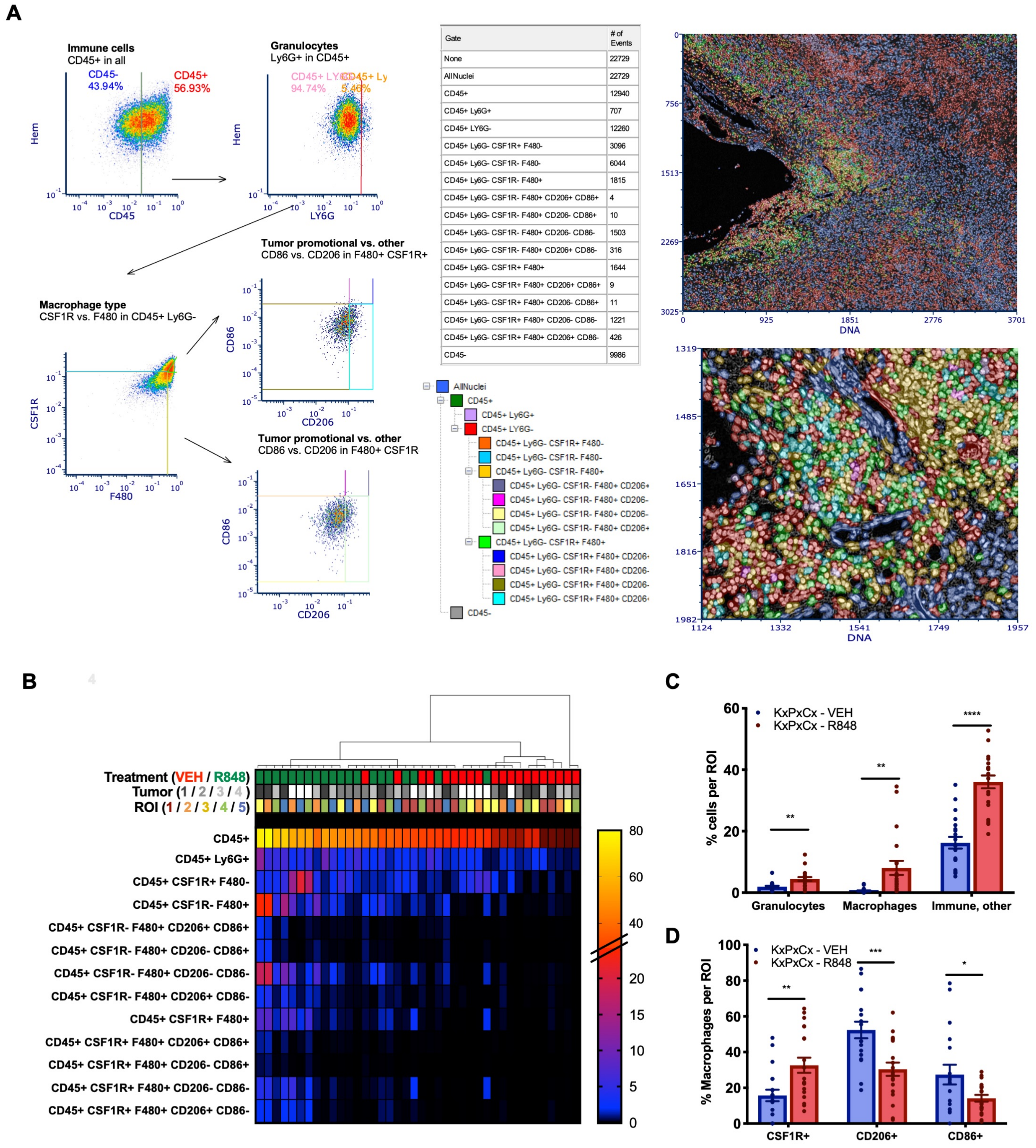

Supplementary Figure 3. Myeloid subanalysis of quantitative multiplex IHC. A separate set of regions representing concentrated areas of immune enrichment within tumor were compared between KxPxCx treated with vehicle versus R848. Staining with markers CD45, Ly6G, CSF1R, F480, CD206, and CD86 was performed. A) Gating strategy and representative results for image cytometry analysis. B) Unsupervised clustering and heatmap analysis, depicted by treatment, sample, region within sample, and frequency of specified populations out of total. C) Frequencies of cell types within vehicle and R848 treated tumor samples, as a percentage of all cells within the ROI. D) Functional markers of macrophage subset, plotted as a percent of macrophages within each ROI. For all analyses, *n* = 20 ROI/group, with 4 tumors/group.

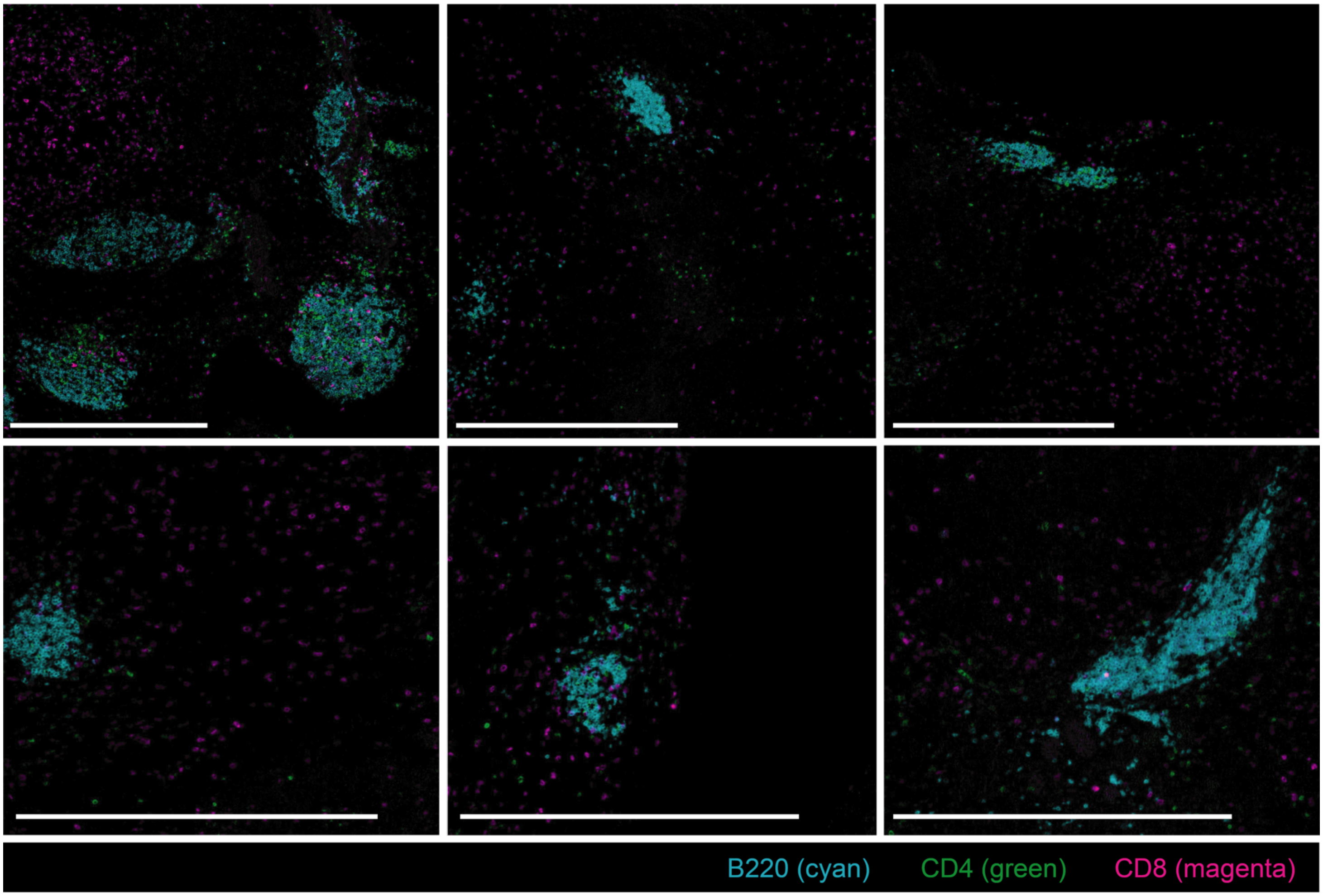

Supplementary Figure 4. IHC of representative intratumoral lymphoid aggregates in R848-treated KxPxCx-derived tumors. B cells are labeled with the marker B220 (cyan), and T cells are labeled with the markers CD4 (green) and CD8 (magenta). Top and bottom rows represent low and high magnification regions respectively, with the scale bar denoting 500 microns in each image.

A

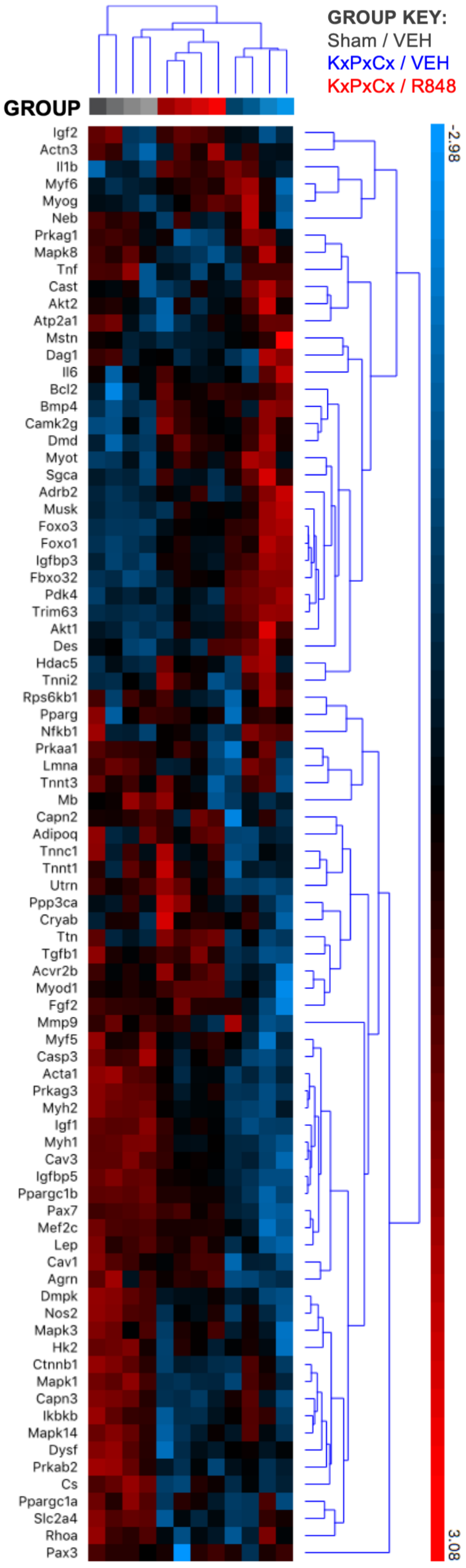

B

	KPC / VEH vs. SHAM / VEH (Fold change)	*p* value	KPC / R848 vs. SHAM / VEH (Fold change)	*p* value	KPC / VEH vs. KPC / R848 (Fold change)	*p* value
Acta1	0.612	0.0004	0.713	0.0024	0.858	0.0319
Actn3	1.036	0.7297	1.137	0.2882	0.911	0.2731
Acvr2b	0.705	0.0227	1.243	0.0843	0.567	0.0068
Adipoq	0.538	0.0945	1.035	0.8942	0.520	0.0053
Adrb2	2.422	0.0663	1.389	0.1148	1.744	0.1715
Agrn	0.657	0.0105	0.924	0.4619	0.711	0.0004
Akt1	2.750	0.0541	1.251	0.2479	2.198	0.0926
Akt2	1.161	0.1818	0.911	0.2804	1.275	0.0826
Atp2a1	1.061	0.5885	0.849	0.2256	1.250	0.0435
Bcl2	1.716	0.0117	1.619	0.0267	1.060	0.6199
Bmp4	1.765	0.0211	1.563	0.0039	1.129	0.4782
Camk2g	1.556	0.0241	1.543	0.0170	1.008	0.9546
Capn2	0.840	0.1073	1.019	0.7900	0.824	0.1344
Capn3	0.751	0.0602	0.622	0.0007	1.208	0.2192
Casp3	0.446	0.0036	0.648	0.0300	0.688	0.0251
Cast	1.351	0.0731	0.982	0.8714	1.375	0.0526
Cav1	0.600	0.0045	0.913	0.3557	0.657	0.0043
Cav3	0.412	0.0002	0.684	0.0025	0.602	0.0054
Cryab	0.966	0.8542	1.455	0.1754	0.664	0.1764
Cs	0.535	0.0007	0.569	0.0068	0.940	0.7242
Ctnnb1	0.800	0.0379	0.728	0.0012	1.098	0.3149
Dag1	1.075	0.7425	0.888	0.2223	1.210	0.4044
Des	1.413	0.0138	1.101	0.3921	1.283	0.0524
Dmd	1.297	0.0170	1.268	0.0028	1.023	0.7528
Dmpk	0.510	0.0008	0.635	0.0017	0.802	0.0528
Dysf	0.752	0.0087	0.631	0.0046	1.190	0.0829
Fbxo32	10.621	0.0016	2.880	0.0069	3.688	0.0052
Fgf2	0.615	0.0315	1.006	0.9554	0.611	0.0609
Foxo1	5.917	0.0326	2.038	0.0061	2.903	0.0736
Foxo3	3.178	0.0106	1.602	0.0001	1.983	0.0387
Hdac5	1.216	0.1834	1.162	0.0456	1.047	0.7349
Hk2	0.676	0.0255	0.704	0.0209	0.960	0.8073
Igf1	0.276	0.0001	0.582	0.0044	0.474	0.0014
Igf2	0.808	0.2901	1.148	0.3904	0.704	0.0017
Igfbp3	6.241	0.0117	2.209	0.0088	2.826	0.0351
Igfbp5	0.062	0.0001	0.247	0.0005	0.252	0.0003
Ikbkb	0.866	0.1464	0.721	0.0035	1.202	0.0895
Il1b	5.594	0.1111	6.421	0.0010	0.871	0.7600
Il6	2.395	0.2518	1.347	0.3791	1.777	0.3825
Lep	0.071	0.0417	0.441	0.1818	0.161	0.0132
Lmna	0.870	0.2989	0.790	0.0039	1.102	0.5158
Mapk1	0.812	0.0316	0.731	0.0001	1.110	0.2370
Mapk14	0.760	0.0365	0.648	0.0043	1.174	0.2850
Mapk3	0.792	0.0299	0.881	0.1377	0.899	0.2910
Mapk8	1.000	0.9976	0.797	0.0013	1.255	0.0476
Mb	0.487	0.0446	0.799	0.5444	0.609	0.2521
Mef2c	0.294	0.0011	0.644	0.0045	0.457	0.0078
Mmp9	0.792	0.4878	0.777	0.1419	1.018	0.9618
Mstn	2.108	0.2720	0.840	0.1379	2.511	0.2141
Musk	2.591	0.0422	1.395	0.0794	1.857	0.1122
Myf5	0.443	0.0265	0.673	0.1207	0.658	0.0667
Myf6	1.717	0.1777	1.441	0.0011	1.191	0.5796
Myh1	0.467	2.77E-05	0.674	0.0008	0.694	0.0078
Myh2	0.466	3.01E-05	0.640	3.81E-05	0.728	0.0038
Myod1	0.387	0.0133	2.357	0.0012	0.164	0.0004
Myog	1.748	0.1993	1.501	0.0759	1.165	0.6710
Myot	1.557	0.0230	1.283	0.0179	1.213	0.1953
Neb	0.967	0.8211	0.872	0.1255	1.109	0.5423
Nfkb1	1.099	0.4134	0.967	0.7458	1.136	0.1361
Nos2	0.524	0.0032	0.631	0.0059	0.831	0.2746
Pax3	0.854	0.4942	0.731	0.4566	1.168	0.6995
Pax7	0.310	0.0001	0.819	0.0704	0.378	0.0015
Pdk4	15.356	0.0084	1.181	0.6444	13.006	0.0090
Pparg	0.901	0.6325	1.009	0.9558	0.893	0.4004
Ppargc1a	0.629	0.0259	0.703	0.0231	0.895	0.5704
Ppargc1b	0.244	8.93E-06	0.585	0.0001	0.417	0.0004
Ppp3ca	0.886	0.2338	1.242	0.1534	0.713	0.0639
Prkaa1	0.870	0.2472	0.900	0.1548	0.967	0.8028
Prkab2	0.755	0.0049	0.727	0.0044	1.038	0.5163
Prkag1	1.049	0.4941	0.826	0.0220	1.269	0.0276
Prkag3	0.121	0.0002	0.324	0.0010	0.375	0.0048
Rhoa	0.891	0.1499	0.857	0.0116	1.040	0.6233
Rps6kb1	1.016	0.8931	0.960	0.5970	1.058	0.6229
Sgca	1.428	0.0212	1.119	0.1903	1.277	0.0911
Slc2a4	0.633	0.0087	0.563	0.0013	1.124	0.5298
Tgfb1	0.718	0.0114	1.036	0.6960	0.693	0.0011
Tnnc1	0.667	0.1066	1.068	0.7925	0.625	0.0930
Tnni2	1.302	0.1503	1.224	0.2906	1.064	0.7706
Tnnt1	0.666	0.0584	1.192	0.5074	0.559	0.0827
Tnnt3	0.935	0.3325	0.941	0.4062	0.993	0.9413
Trim63	20.389	0.0085	1.957	0.1026	10.417	0.0108
Ttn	0.924	0.2749	1.174	0.0222	0.787	0.0009
Utrn	0.782	4.96E-05	1.040	0.5110	0.752	0.0031

Supplementary Figure 5. Targeted array of myopathy- and myogenesis-associated genes in skeletal muscle from control mice and tumor-bearing mice with and without R848 therapy. A) Unsupervised clustering analysis of all array transcripts, normalized to mean expression within transcript and scaled by standard deviation. B) Fold regulation values and P values for all transcripts, with ddCT values normalized to 5 control transcripts (*Actb, B2m, Gapdh, Gusb*, and *Hsp09ab1*).

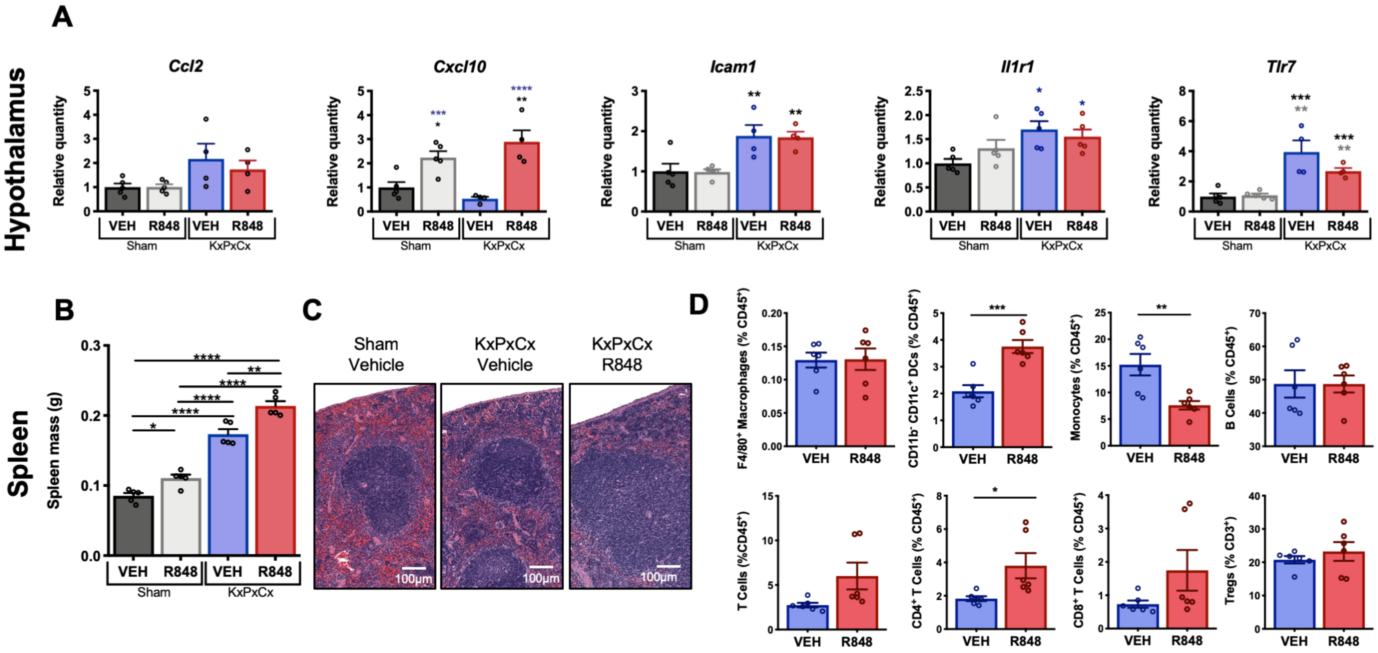

Supplementary Figure 6. Extended data on cachexia molecular physiology. A) Hypothalamic gene expression of inflammatory transcripts *Ccl2, Cxcl10*, *Icam1*, *Il1r1*, and *Tlr7* (n=5/group). B) Spleen mass at necropsy for sham-operated and KxPxCx-engrafted mice treated with R848 or vehicle (n=5/group). C) Representative splenic H&E, imaged at 20x, demonstrating progressive inflammatory responses to tumor burden and reactive lymphoid hyperplasia following treatment with R848. D) Flow cytometric analysis of splenic immune populations in tumor-bearing animals allocated to vehicle (VEH) or R848 (*n*=6/group). *, *P*<0.05; **, *P*<0.01; ***, *P*<0.001.

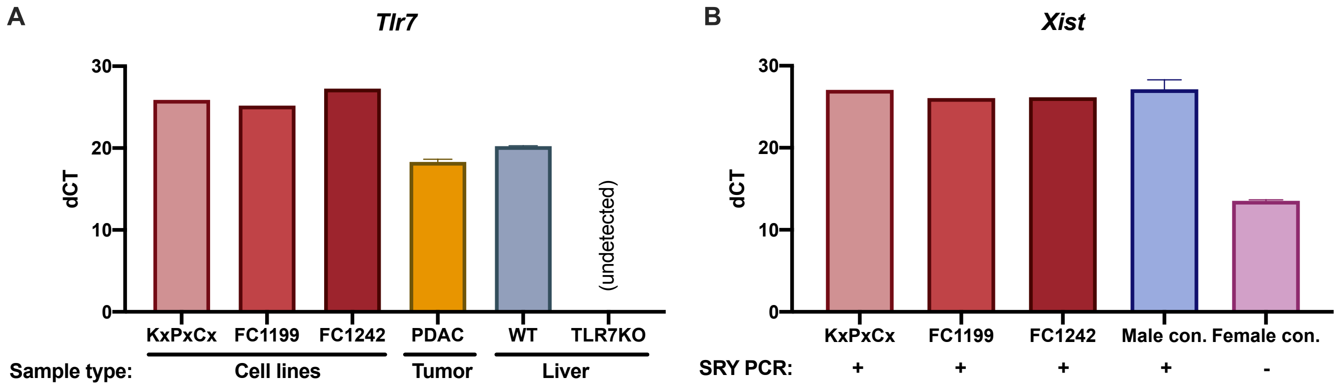

Supplementary Figure 7. Extended data on cell lines. Gene expression is depicted as dCT relative to 18S, such that higher values correspond to lower levels of relative expression. A) *Tlr7* expression in cell lines KxPxCx, FC1199, FC1242, a KxPxCx-derived PDAC tumor, and livers from control WT and TLR7KO mice. B) *Xist* gene expression and SRY PCR results in cell lines KxPxCx, FC1199, FC1242, male control liver, and female control cell line SIM-A9.
