# Supplementary material for: The TLR7/8 agonist R848 remodels tumor and host responses to promote survival in pancreatic cancer
Source: Nat Commun. 2019 Oct 15;10:4682. doi: 10.1038/s41467-019-12657-w (PMC6794326; doi:10.1038/s41467-019-12657-w)
Supplement: Supplementary file 2 — Description of Additional Supplementary Files [file 41467_2019_12657_MOESM2_ESM.docx]

Description of Additional Supplementary Files

**Supplementary Movie 1:** Behavior and appearance of a KxPxCx-engrafted mouse with cachexia on day 14, as compared to KxPxCx-engrafted mice treated with either burst or continuous R848 on day 33.
