# Supplementary material for: The TLR7/8 agonist R848 remodels tumor and host responses to promote survival in pancreatic cancer
Source: Nat Commun. 2019 Oct 15;10:4682. doi: 10.1038/s41467-019-12657-w (PMC6794326; doi:10.1038/s41467-019-12657-w)
Supplement: Supplementary file 4 — Reporting Summary [file 41467_2019_12657_MOESM4_ESM.pdf]

nature research

Corresponding author(s): Daniel L. Marks

Last updated by author(s): July 21, 2019

Reporting Summary

Nature Research wishes to improve the reproducibility of the work that we publish. This form provides structure for consistency and transparency in reporting. For further information on Nature Research policies, see Authors & Referees and the Editorial Policy Checklist.

Statistics

For all statistical analyses, confirm that the following items are present in the figure legend, table legend, main text, or Methods section.

n/a	Confirmed	
☐	☒	The exact sample size (*n*) for each experimental group/condition, given as a discrete number and unit of measurement
☐	☒	A statement on whether measurements were taken from distinct samples or whether the same sample was measured repeatedly
☐	☒	The statistical test(s) used AND whether they are one- or two-sided<br>*Only common tests should be described solely by name; describe more complex techniques in the Methods section.*
☐	☒	A description of all covariates tested
☐	☒	A description of any assumptions or corrections, such as tests of normality and adjustment for multiple comparisons
☐	☒	A full description of the statistical parameters including central tendency (e.g. means) or other basic estimates (e.g. regression coefficient) AND variation (e.g. standard deviation) or associated estimates of uncertainty (e.g. confidence intervals)
☐	☒	For null hypothesis testing, the test statistic (e.g. *F*, *t*, *r*) with confidence intervals, effect sizes, degrees of freedom and *P* value noted<br>*Give P values as exact values whenever suitable.*
☒	☐	For Bayesian analysis, information on the choice of priors and Markov chain Monte Carlo settings
☒	☐	For hierarchical and complex designs, identification of the appropriate level for tests and full reporting of outcomes
☒	☐	Estimates of effect sizes (e.g. Cohen's *d*, Pearson's *r*), indicating how they were calculated

Our web collection on statistics for biologists contains articles on many of the points above.

Software and code

Policy information about availability of computer code

Data collection	Raw scans at 20x magnification were obtained as .svs files for all multiplexed histology using Leica Aperio software, with regions of interest (ROIs) defined in ImageScope software. For locomotor activity and body temperature readouts, counts were measured by MiniMitter VitalView software and exported as ASCII format for further analysis.
Data analysis	For multiplex immunohistochemistry analysis, ROIs were aligned for all markers using the SURF algorithm in MATLAB. These registered regions were imported into FIJI-Image J to extract AEC signal from background using color deconvolution, and generate single cell segmentation masks. Images for markers of interest were merged in FIJI-Image J for visualization purposes. Cell Profiler was used to measure the mean intensity of each marker in each segmented cell. This output data was imported into FCS Express 6.0 Image Cytometry, and manually gated with use of picture plots to validate the expression of each cell as true positive or true negative for each marker. Unsupervised clustering analyses and heat map visualization were performed in SPSS (IBM) and Orange (University of Ljubljana). All other statistical analyses were performed using Prism 8.0 software.

For manuscripts utilizing custom algorithms or software that are central to the research but not yet described in published literature, software must be made available to editors/reviewers. We strongly encourage code deposition in a community repository (e.g. GitHub). See the Nature Research guidelines for submitting code & software for further information.

Data

Policy information about availability of data

All manuscripts must include a data availability statement. This statement should provide the following information, where applicable:

- Accession codes, unique identifiers, or web links for publicly available datasets
- A list of figures that have associated raw data
- A description of any restrictions on data availability

All data associated with this study are available in the main text or the supplementary materials. There are no restrictions on data availability.

Field-specific reporting

Please select the one below that is the best fit for your research. If you are not sure, read the appropriate sections before making your selection.

☒ Life sciences ☐ Behavioural & social sciences ☐ Ecological, evolutionary & environmental sciences

For a reference copy of the document with all sections, see nature.com/documents/nr-reporting-summary-flat.pdf

Life sciences study design

All studies must disclose on these points even when the disclosure is negative.

Sample size	The sample size was determined based on prior experience with the KxPxCx model for cachexia studies, such that we would be able to readily distinguish sham-operated animals from tumor-bearing animals for a wide variety of parameters including behavior and gene expression in cachexia-sensitive tissues, and able to compare the effects of R848 with respect to both tumor-bearing and sham-operated controls. Sample sizes for FC1242 and FC1199 were determined by similar metrics, using pilot data comparing orthotopic implantation of 5 KPC-derived cell lines to test magnitude and variance of cachexia-related outcomes. Because we anticipated cachexia measurements to have more variability and lower magnitude of difference than tumor measurements, power analyses were not performed on the basis of tumor mass or composition.
Data exclusions	Our a priori policy was to exclude animals and their samples from analysis (1) if tumor inoculation was unsuccessful, or (2) if random events over the course of the study with confounding potential would put the animal in a biologically distinct category unrelated to their tumor status or treatment allocation (such as treatment administration complications resulting in large volume bleeding, a cage disruption such as water leaking, etc.)
Replication	To ensure the robustness of the effects on tumor progression and cachexia, studies performed with the primary cell line KxPxCx were replicated in a total of 4 studies across 2 routes of administration. To ensure that the effect was not specific to an uncommon vulnerability in KxPxCx, we additionally tested the effects of R848 using two additional epithelial clones, FC1242 and FC1199. The commonalities and variations between these epithelial cell lines with respect to tumor response and cachexia are discussed in the study.
Randomization	Randomization of groups was performed to ensure variables related to cachexia were balanced across tumor and sham-operated animals, using the covariates initial body weight, initial lean mass, initial fat mass, and initial food intake. Animals were further randomized for these covariates when multiple treatment groups were present, such that cachexia-related parameters were expected to be evenly distributed between groups. T-tests (for two groups) or ANOVA (for 3+ groups) were performed within studies for all relevant variables to ensure no significant differences were present at baseline for any of the aforementioned characteristics, such that changes at endpoint could be attributed to the disease state or treatment group rather than baseline differences.
Blinding	Blinding was performed for any qualitative analysis, including histology scoring and interpretation. In animal models, investigators were aware of which treatment they were delivering to subjects, and whether the subject was tumor-bearing or sham-operated, but all collected data were quantitative in nature during this phase. Gene expression studies and samples acquired from these studies were coded and blinded for downstream gene expression analyses.

Reporting for specific materials, systems and methods

We require information from authors about some types of materials, experimental systems and methods used in many studies. Here, indicate whether each material, system or method listed is relevant to your study. If you are not sure if a list item applies to your research, read the appropriate section before selecting a response.

Materials & experimental systems

n/a	Involved in the study
☐	☒ Antibodies
☐	☒ Eukaryotic cell lines
☒	☐ Palaeontology
☐	☒ Animals and other organisms
☒	☐ Human research participants
☒	☐ Clinical data

Methods

n/a	Involved in the study
☒	☐ ChIP-seq
☐	☒ Flow cytometry
☒	☐ MRI-based neuroimaging

Antibodies

Antibodies used	Antibodies, with corresponding catalog numbers and manufacturers, are described in Supplementary Methods under headings Materials Used for Flow Cytometry and Antibodies Used for IHC.
Validation	For mIHC, antibodies were validated previously in a separate study using a panel of healthy tissue and a variety of solid tumors. Within-study validation was performed on this multiplex using spleen and liver as positive control tissues representing the majority of cell lineages present within tumor immune microenvironment, in addition to validation steps such as IHC and

Western performed by the manufacturer. Details of these validation steps are present on the websites for each antibody, and are not elaborated on here because of the large number of antibodies used in this study (36).

Eukaryotic cell lines

Policy information about cell lines

Cell line source(s)	For these studies, we used three epithelial clones derived from the KPC pancreatic cancer model, which is described in our methods. KxPxCx cells were a kind gift of Dr. Elizabeth Jaffee of Johns Hopkins University, and FC1242 and FC1199 were generously provided by Dr. David Tuveson of Cold Spring Harbor Laboratory.
Authentication	We have not personally authenticated the cell lines in these studies using a professional service; however, they form tumors consistent with moderately-to-poorly differentiated pancreatic ductal adenocarcinoma in vivo.
Mycoplasma contamination	All cell lines in this study undergo routine mycoplasma testing, and have been consistently negative. They additionally have undergone PCR screening by IDEXX Bioresearch for 40 microbial and viral contaminants, for which they were negative.
Commonly misidentified lines (See ICLAC register)	We did not use any commonly misidentified lines in this paper.

Animals and other organisms

Policy information about studies involving animals; ARRIVE guidelines recommended for reporting animal research

Laboratory animals	Male C57BL6/J mice (JAX, cat. #000664) and TLR7KO mice backcrossed to the C57BL6/J background (JAX, cat. #008380) were used in these studies, with implantation occurring at 8-9 weeks of age.
Wild animals	*Provide details on animals observed in or captured in the field; report species, sex and age where possible. Describe how animals were caught and transported and what happened to captive animals after the study (if killed, explain why and describe method; if released, say where and when) OR state that the study did not involve wild animals.*
Field-collected samples	*For laboratory work with field-collected samples, describe all relevant parameters such as housing, maintenance, temperature, photoperiod and end-of-experiment protocol OR state that the study did not involve samples collected from the field.*
Ethics oversight	Animal studies were performed in accordance with the Oregon Health and Science University IACUC, with principles following the NIH Guide for the Care and Use of Laboratory Animals.

Note that full information on the approval of the study protocol must also be provided in the manuscript.

Flow Cytometry

Plots

Confirm that:

- [x] The axis labels state the marker and fluorochrome used (e.g. CD4-FITC).
- [x] The axis scales are clearly visible. Include numbers along axes only for bottom left plot of group (a 'group' is an analysis of identical markers).
- [x] All plots are contour plots with outliers or pseudocolor plots.
- [x] A numerical value for number of cells or percentage (with statistics) is provided.

Methodology

Sample preparation	Tumor-bearing mice were perfused with PBS to clear tissue of vascular immune cells. Tumors were harvested in DMEM on ice, minced, and enzymatically digested in DMEM containing 1.0 mg/mL Collagenase IV (Gibco, #17104-019), 1.0 mg/mL Soybean Trypsin Inhibitor (Gibco; #17075-029), and 50 U/mL DNAse 1 (Roche; #10104159001). Samples were incubated for 30 minutes at 37 degrees C with agitation at 125 rpm, filtered through 100μM cell strainers, centrifuged at 400g for 5 minutes at 4 degrees C, then resuspended and immediately stained for flow cytometry.
Instrument	BD Fortessa
Software	Flow cytometry data was analyzed using FlowJo software.
Cell population abundance	This data is provided in Fig. 1e.
Gating strategy	The gating strategy, with specific cutoffs for gates, is included in Fig. 1c.

- [x] Tick this box to confirm that a figure exemplifying the gating strategy is provided in the Supplementary Information.
